# Acidic and Hypoxic Microenvironment in Melanoma: Impact of Tumour Exosomes on Disease Progression

**DOI:** 10.3390/cells10123311

**Published:** 2021-11-25

**Authors:** Zaira Boussadia, Adriana Rosa Gambardella, Fabrizio Mattei, Isabella Parolini

**Affiliations:** 1 National Center for Drug Research and Evaluation, Istituto Superiore di Sanità, Viale Regina Elena 299, 00161 Rome, Italy; zaira.boussadia@iss.it; 2 Department of Oncology and Molecular Medicine, Istituto Superiore di Sanità, Viale Regina Elena 299, 00161 Rome, Italy; adriana.gambardella@guest.iss.it; 3 Department of Translational Medical Sciences, Center for Basic and Clinical Immunology Research (CISI), University of Naples Federico II, 80131 Naples, Italy

**Keywords:** tumour exosomes, melanoma, acidic and hypoxic microenvironment, cancer associated fibroblasts, biomarkers

## Abstract

The mechanisms of melanoma progression have been extensively studied in the last decade, and despite the diagnostic and therapeutic advancements pursued, malignant melanoma still accounts for 60% of skin cancer deaths. Therefore, research efforts are required to better define the intercellular molecular steps underlying the melanoma development. In an attempt to represent the complexity of the tumour microenvironment (TME), here we analysed the studies on melanoma in acidic and hypoxic microenvironments and the interactions with stromal and immune cells. Within TME, acidity and hypoxia force melanoma cells to adapt and to evolve into a malignant phenotype, through the cooperation of the tumour-surrounding stromal cells and the escape from the immune surveillance. The role of tumour exosomes in the intercellular crosstalk has been generally addressed, but less studied in acidic and hypoxic conditions. Thus, this review aims to summarize the role of acidic and hypoxic microenvironment in melanoma biology, as well as the role played by melanoma-derived exosomes (Mexo) under these conditions. We also present a perspective on the characteristics of acidic and hypoxic exosomes to disclose molecules, to be further considered as promising biomarkers for an early detection of the disease. An update on the use of exosomes in melanoma diagnosis, prognosis and response to treatment will be also provided and discussed.

## 1. Introduction

Cutaneous melanoma (CM) originates from melanocytes resident in the skin and accounts for more than 90% of all melanoma cases registered in the United States, including uveal or mucosal melanoma [1]. Globally, malignant melanoma accounts for 1% of skin tumours and causes of 60% of the mortality due to skin cancers [2]. CM is characterized by high aggressiveness and a remarkable resistance to chemotherapeutic drugs. The disease evolves quite easily from a primary to a metastatic form when the cells leave the epithelium of the epidermis and enter into the adjacent tissues [3]. Despite the clinical benefits of immune checkpoint inhibitors in treating melanoma [4], the incidence and death rate of this disease continues to increase [5]. Therefore, an in-depth comprehension of the mechanisms underlying the development of melanoma is demanded.

Nowadays, melanoma is defined as a multi-factorial disease arising from an interaction between environmental risk factor exposure (ultraviolet exposure) and genetic susceptibility. However, TME also plays a significant role in the disease development, as largely described in the past few years [6].

TME represents a hostile milieu in which tumour cells are favoured over non-tumour cells that cannot adapt. The main determinants of TME are acidity and hypoxia. To support the high proliferation rate, tumour cells are forced to reprogram their metabolism and to increase the rate of glucose uptake, through the up-regulation and the enhanced activity of glucose transporters [7,8], leading to a high production of lactate and intracellular accumulation of protons (Warburg Effect). To counteract this increased proton level, tumour cells overexpress vacuolar ATPase (V-ATPase), Na+/H+ exchanger (NHE), monocarboxylate transporters (MCTs) and carbonic anhydrase [9], that actively export protons that accumulate into an extracellular microenvironment [10]. As a result, the extracellular pH (pHe) decreases to as low as 6.5, a value that is toxic to normal cells, whereas cancer cells adapt and survive [10,11,12].

In this context, the increased metabolic need of tumour cells cannot be accomplished by higher oxygen consumption, due to the altered angiogenesis and to the shift from oxidative phosphorylation (OXPHOS) in the mitochondria towards glycolysis [13]. Glycolysis produces L-lactate, which is responsible for the pH reduction. Hypoxia can also induce proteomic changes that initiate cell cycle arrest, differentiation, necrosis, apoptosis [14], and may also stimulate tumour growth, invasion, and metastasis [15].

In these environmental conditions, the tumor mass increase is also due to an impaired immune surveillance. In fact, acidity was described to shorten the lifespan or inhibit the proliferation of CD4+ and CD8+ Cytotoxic T Lymphocytes (CTLs), Dendritic cells (DCs), and Natural Killer (NK) cells [16], whereas hypoxia could limit the level of M2 macrophages, T cells, and NKs, thus negatively affecting the immune system response and the consequent cancer aggressiveness [17].

In general, tumour cells use an indirect and vesicles-based mechanism to affect either the growth of tumour mass or the immune cells surveillance. This mechanism takes advantage of a plethora of extracellular vesicles (EVs) constitutively and abundantly secreted from tumour cells, that are endowed with proteins, lipids, and nucleic acids (DNA, mRNA, cirRNA, miRNAs, and lncRNAs) which can be shuttled between neighbouring and distant cells, in turn altering the physiological functions of recipient cells.

EVs in general are represented by a heterogeneous subsets of vesicles including small EVs (sEVs) (30–150 nm), microvesicles (200–1000 nm), apoptotic bodies (>1000 nm) of different biogenesis, release mechanisms and biochemical properties.

To date, a big debate is still ongoing among scientists to convey in standardized procedures for the isolation and characterization of distinct subtype of vesicles and guidelines were reported [18]. In the meantime, the available procedures allow the separation of vesicles based on size, regardless of their endosomal or plasma membrane origin and a simplified nomenclature was adopted in the literature to refer to smaller EVs (sEVs) (<200 nm) and larger EVs (>200 nm). sEVs include among others a subset of endolysosomal-origin vesicles named exosomes (Exo), that have attracted much attention as important carriers of intercellular messages in TME and as a potential diagnostic tool in cancer [19,20,21].

Given the strong association between environmental hypoxia, acidity, and melanoma progression, we will provide an overview of the recent studies on the impact of these conditions in melanoma development, and on the role of Mexo in evading the immune system surveillance, as well as in promoting tumour progression. Finally, we will highlight molecules (proteins, miRNAs) found upregulated in Exo under acidic and hypoxic conditions, in cell culture or patient plasma and we will discuss their potential use as biomarkers.

## 2. Role of Acidic and Hypoxic Microenvironment in Melanoma

The extracellular acidosis was described to play combined roles in melanoma development, either at tumour cell level or in the crosstalk between tumour and stroma, resulting in the tissue remodelling necessary to support the local invasion. At tumour cell level, acidity was described to inhibit mTOR signalling and downregulate energy-consuming metabolism [22], leading to a decrease in cell growth. This could explain the enhanced resistance to chemotherapeutic agents, targeting fast-growing cancer cells [23] and the subsequent possible repopulation following chemotherapy. An additional role played by acidosis could regard the dedifferentiation of cancer cells, i.e., the malignant progression of cancer cells through the induction of a cancer stem cell phenotype [24]. Indeed, cancer stem cells entail the ability to self-renew and differentiate in a variety of tumour cell subpopulations, as well as to enter in a quiescent state responsible of chemo- and radio resistance [25,26]. Moreover, in melanoma it was demonstrated that acidity triggers epithelial–mesenchymal transition (EMT) [27], a step of carcinoma progression, during which the cells acquire properties that promote metastases [28]. Among these properties, cells upregulate mesenchymal markers (N-cadherin, Vimentin) and transcription factors (Twist, NF-KB) and downregulate E-cadherin expression.

Acidosis can contribute to tumour advancement by inducing the expression of degrading enzymes necessary for the disruption of extracellular matrix, a prerequisite for tumour cell invasion, metastasis and angiogenesis. In line with this, extracellular acidosis induced MMP-9 expression in mouse metastatic melanoma cells [29], and the upregulation of the proteolytic enzymes MMP-2, cathepsin B, and cathepsin L [30], in turn enhancing the invasive potential of human melanoma cells and the ability to develop pulmonary metastases.

The generation of a hypoxic environment generally occurs in advanced cancers and implies the activation of hypoxia-inducible factors (HIFs) including HIF1, HIF2 and HIF3 [31,32], in turn promoting the adaptation and selection of both cancer and stromal cells able to support cancer progression.

In a pooled dataset of melanoma patients, a positive correlation between the presence of hypoxia within the tumour mass and a poor prognosis for these patients was found [33]. Hypoxia was described to drive metastatic progression by promoting a switch from a proliferative to an invasive phenotype, in that the exposure of proliferative melanoma cells to hypoxic microenvironments was sufficient, in a HIF1α-dependent manner, to downregulate melanocytic marker expression and to increase their invasive potential [34]. This can occur through nuclear translocation of HIF-1α, which in turn promotes the upregulation and stabilization of Snail and Twist, resulting in the cadherin switching [35,36].

In line with this, it was reported the overexpression of HIF-1α in biopsies derived from skin cancer and uveal melanoma patients [37]. Other authors hypothesized that HIF-1α was also upregulated in metastatic tissue with respect to the primary tumours, and, its expression correlated with high expression of proliferative and vascular markers [38]. HIF-1α is responsible for the upregulation of miR-210, together with miR-224, miR-452, and miR-218, causing an increase in BNIP3 and ATF3 [39]. miR-210 plays an important role in the cell cycle arrest and is able to support the tumour cell growth even in hypoxic conditions [40]. Interestingly, miR-210 regulates the susceptibility of tumour cells to lysis by CTLs by targeting PTPN1, HOXA1 and TP53I11 [41]. In fact, the knock down of miR-210 restores the sensitivity of tumour cells to CTL lysis. Thus, this miRNA represents a link between hypoxia and immune escape. Accordingly, miR-210 was found upregulated in plasma of metastatic melanoma patients [42].

The other active HIF-1 subunit, HIF-2α, was not extensively studied in melanoma and the data are often inconclusive. However, there is a positive correlation between poor prognosis and over-expression of HIF-2α as well as vascular endothelial growth factor (VEGF) in patients affected by nodular malignant melanoma [43].

Another way to react to oxygen lack is the promotion of neo-angiogenesis. In melanoma some miRNAs are known to regulate the vascular formation: miR-1908, miR-199a-5p, and miR-199a-3p. These miRNAs convergently target apolipoprotein E (ApoE) and the heat shock factor DNAJA4 thus promoting invasion and metastatic endothelial recruitment [44].

These data collectively suggest the important role played by the melanoma microenvironment in the development of the disease.

## 3. How Acidity and Hypoxia Modulate Melanoma Progression through Mexo

### 3.1. Characteristics of Mexo Released in Acidic and Hypoxic TME

Exo are nanosized (30–120 nm) vesicles able to exchange their content between neighbouring cells within TME or cells located at distant target organs. In general, the protein content of Exo reflects primarily the biogenesis pathway, i.e., members of the tetraspanin family (CD9, CD63, CD8), members of the ESCRT complex (TSG101, Alix) and Heat Shock Proteins (HSP60, HSP70, HSP90) [45]. Furthermore, Exo also contain some specific proteins that mirror the cell type from which they originate, such as epithelial cell adhesion molecule (EpCAM) [46,47], melanoma antigen recognized by T cells 1 (Mart-1) [48,49], human epidermal receptor (HER) (breast cancer, and pancreatic cancer origin) [50,51,52].

Mexo were described to vehiculate functionally active molecules involved in angiogenesis and in tumour growth and metastasis formation, such as interleukin (IL)-6, vascular endothelial growth factor (VEGF) and different metalloproteinase (MMPs) [53,54], as well as miRNAs, such as miR-494 [55], miR-9 [56], miR-125b [57], miR-155-5p [58], miR-91, Let-7a, Let-7i [59], and miR-222 [60]. Interestingly, some of these miRNAs are enriched in Exo secreted by metastatic cell lines [55,60] compared to primary tumour cells, highlighting these vesicles as a potential prognostic tool.

In melanoma, it has recently been described that various stress conditions modify Mexo cargo to promote or suppress existing signalling pathways and to activate new pathways, ultimately triggering a unique cell-specific response pattern in target cells [61]. Within TME, hypoxia and acidity represent the main stress conditions deeply influencing Exo secretion and cargo. Walbrecq et al. recently reported that hypoxic cells released a higher amount of Mexo than normoxic cells, in turn promoting invasion of melanoma cells, mediated by miR-1290 [62]. In addition, they found a signature in hypoxic Mexo, of proteins specifically correlated with a poor prognosis (AKR7A2, DDX39B, EIF3C, FARSA, PRMT5, VARS) and with a decreased survival (HNRNPL, HNRNPK, RAN) in melanoma patients. Interestingly, most of these proteins were found to promote proliferation, migration, and invasion in various tumours or to be involved in drug resistance (Table 1). In line with this, hypoxic Mexo were able to enhance the migration and invasion in target normoxic melanoma cells. Hypoxia was described to affect also miR-494-5p, miR-4497, and miR-513a-5p content of Mexo [63]. The functional analysis of the upregulated miRNAs indicated the cellular processes likely involved in melanoma progression such as proliferation, drug resistance and modification of the tumour microenvironment, including immunosuppression [63]. These factors all contribute to a more highly invasive potential of melanoma cells.

A recent study describes how Mexo may also be involved in the formation of the acidic microenvironment by the induction of a metabolic reprogramming in target stromal fibroblasts. This involves an increase in aerobic glycolysis and a decrease in OXPHOS, mediated by the delivery of miR-210 and miR-155 [64].

The extracellular acidity is a condition that tumour cells create to select themselves to grow and evolve toward a metastatic phenotype [30,65], likely through a mechanism based on the intercellular exchange of Exo. In fact, acidic TME is able to up modulate exosome biogenesis and secretion as reported in several human metastatic melanoma cell lines [66,67,68]. Interestingly, the increase in the acidic Mexo secretion does occur only at specific melanoma stages, when it is necessary the spread of newly acquired information to sustain the advancement of melanoma [66].

Moreover, the protein cargo of acidic Mexo is enriched with respect to Mexo in proteins related to metastatic processes such as HRAS, GANAB, CFL2, HSP90B1, HSP90AB1, GSN, HSPA1L, NRAS, HSPA5, TIMP3, HYOU1 [66] (Table 1). Interestingly, the molecules upregulated both in acidic and hypoxic Mexo [62] were analysed by PrognoScan and were found correlated with a poor prognosis. Besides, this molecule profile was also detected in biopsies of melanoma patients with a poor prognosis, thus representing a specific signature stage-disease related [66]. In line with this study, some of these proteins (GNS, CFL, HSP90AB1) were found upregulated in Exo derived from plasma of melanoma patients when compared to healthy donors [69]. These results suggest that acidic Mexo are carriers of specific molecules associated with the advanced stage of the disease, and therefore they can be considered a potential reservoirs of prognostic biomarkers. 

**Figure 1:**
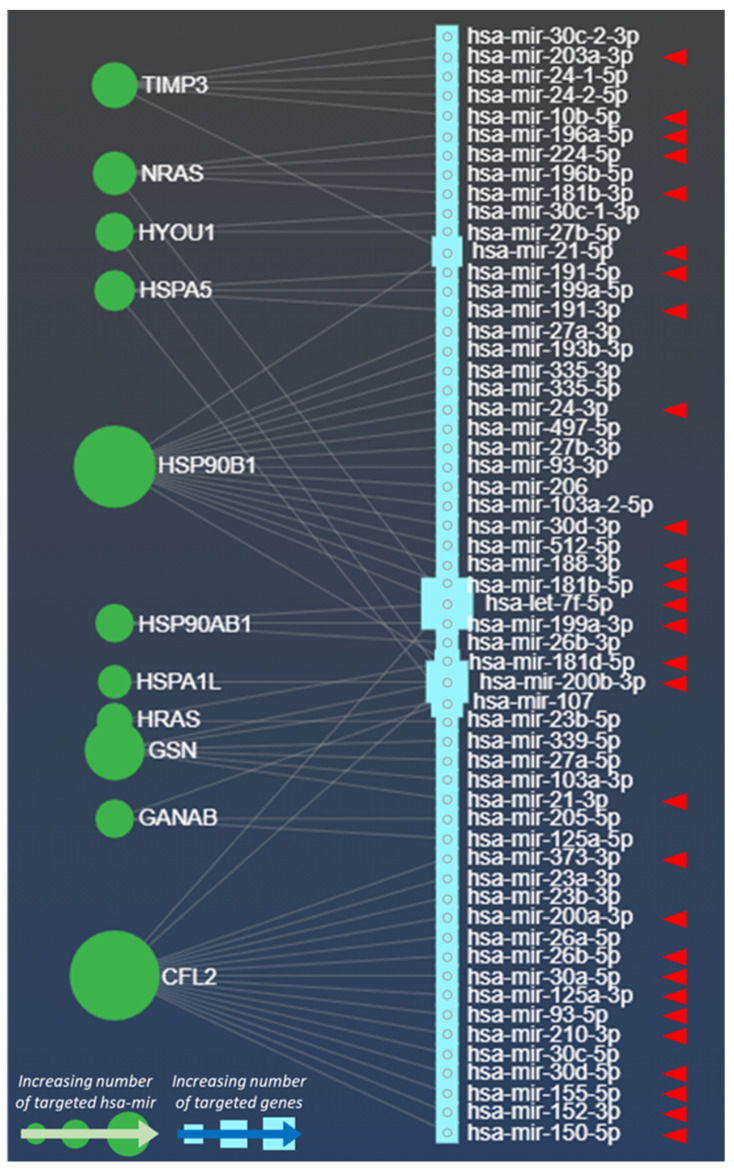
Bipartite network between hypoxic miRNAs and acidic-driven Mexo proteins. These miRNAs are related to hypoxia, and were generally described within Exo, but not within hypoxic Exo. The network (grey lines) displays hypoxia-associated miRNAs (hsa-mir, cyan squares; *n* = 57) targeting the indicated melanoma metastatic genes (green circles) whose expression was found enriched in acidic Mexo (summarized in Table 1). The red arrowheads depict miRNAs experimentally involved in melanoma progression, metastatic expansion and drug therapy resistance (summarized in Table 2). This bipartite network has been generated by interrogating the publicly available database miRNet (https://www.mirnet.ca, accessed on 30 March 2021; see Data Availability Statement for further details) within the exosome dataset (*n* = 1250 hsa-mir) and the indicated genes.

Much evidence from literature has revealed the presence of different miRNAs in tumour Exo, including melanoma. Several of these miRNAs can also act as oncomirs or oncosuppressors or are capable to induce immune escape events [70,71]. Based on these premises, we wondered whether the proteins found in acidic Mexo by Boussadia and co-Workers [66] could be targeted by miRNAs involved in hypoxic processes. To this purpose, we exploited the miRNet database (www.mirnet.ca, accessed on 30 March 2021; see Data Availability Statement for further details), where a specific dataset describing the miRNAs internalized in Exo (*n* = 1250) is publicly available (Figure 1). We generated a miRNA-protein association network (bipartite network) displaying which protein is being targeted by which miRNA. Remarkably, we found that HRAS, GANAB, CFL2, HSP90B1, HSP90AB1, GSN, HSPA1L, NRAS, HSPA5, TIMP3, HYOU1 molecules are targeted by several miRNAs found in Exo (Figure 1, grey lines). Interestingly, these miRNAs are also directly involved in hypoxia [72] and in multiple processes related to melanoma advancement, such as proliferation, progression, metastatic expansion, immunosuppression, and drug resistance (Figure 1, *n* = 57). However, experimental evidences on the exosomal expression of these miRNAs in a hypoxic context are lacking.

Moreover, the involvement of some of these miRNAs in tumor progression processes is also corroborated by significant experimental evidence (Figure 1, red arrowheads), detailed in Table 2 (*n* = 28 hsa-mir), thus underlining the pro-tumoural role of the majority of hypoxia-associated miRNAs displayed in our protein-miRNA network (Figure 1, red arrowheads; Table 2).

Strikingly, part of these miRNAs are also recognised as oncomirs (Table 2). For instance, the oncomir mir-21-3p, targeting the GSN gene (Figure 1) drives to an augmented melanoma invasiveness by decreasing the MMP3 inhibitors in vivo. This oncomir has also been found to sustain melanoma metastatic expansion by promoting the insurgence of NRAS and BRAF mutation and by increasing the expression of L1CAM [73,74] (Table 2). Another oncomir (has-mir-203a-3p) was found linked to stemness and to induce melanoma progression in vivo. Sahranavardfard and co-workers suggest that this oncomir can be used as an early marker for melanoma metastasis detection [75]. Of note, this network depicts how many times a gene is targeted by hsa-miR. For example, CFL2 and HSP90B1 appear to be the most targeted genes by these miRNAs (both by 17 has-miR, Figure 1), whereas HSPA1L is the less targeted with only one hsa-mir (Figure 1).

The observations obtained from our protein-miRNA network (Figure 1) indicate that acidic Mexo contain proteins that can be targeted by miRNA involved in the hypoxia-induced melanoma progression and metastatic expansion. In addition, the expression of almost all proteins upregulated in acidic Mexo (Table 1), is described to be enhanced also in hypoxia [76,77,78,79,80,81]. These considerations may unveil the existence of shared molecules and molecular pathways through an EVs-based mechanism in acidic and hypoxic conditions that could cooperate in favouring tumour progression. However, to validate such hypothesis further studies based on the expression levels of miRNAs listed in Table 2 within hypoxic Mexo will be required.

Taken together, all these proofs of evidence highlight the importance of Mexo secreted from the hypoxic and acid core of the tumours in the establishment of the favourable conditions that sustain the spread of melanoma cells.

### 3.2. Mechanisms of Progression and Metastatic Spread

The study of the molecular content of acidic Mexo shed light on the molecular mechanisms involved in melanoma progression. In fact, a recent study evidenced the release of acidic Mexo containing a large number of molecules regulating several processes involved in cell transformation, such as proto-oncogenes (HRAS, NRAS), metalloprotease (TIMP3), heath shock protein isoforms (HSP90AB1, HSP90B1, HSPAIL, HSPA5), enzymes (GANAB) involved in protein folding and in the control of endoplasmic reticulum and actin-binding proteins (GSN, CFL2) [66]. This release was found to occur at a specific stage of melanoma progression when exposed to an acidic pressure and to affect the behaviour of tumour cells not exposed to acidic pH. This led us to hypothesize that also in vivo, i.e., within the heterogenic tumour mass, exosomes released by some cells in response to acidification might influence the behaviour of neighbouring cells, through a continuous and abundant vesicle intercellular transfer. Such a transfer can occur through a membrane-fusion mechanism favoured by a higher sphingomyelin/ganglioside GM3 (N-acetylneuraminylgalactosylglucosylceramide) exosomal lipid content induced by the acidic pressure [68].

Among neighbouring cells, stromal cells can be reprogrammed to favour tumour growth and diffusion [82,83]. An example is represented by cancer associated fibroblasts (CAFs), a particular type of stromal cell commonly found in the TME [84,85,86]. CAFs can be considered cells negative for epithelial, endothelial and leukocyte markers with an elongated morphology and lacking the mutations found within cancer cells [87].

Studies performed in human tissues described the progressive changes in the fibroblastic stroma, with an expansion of fibroblasts circumscribing early or premalignant lesions. The role of such fibroblasts could be the maintenance of the stromal architecture and connections within TME [52] and also the suppression of EMT and the arrest in the G1/S phase of the cell cycle, in turn inhibiting the melanoma development at early stages [85,88]. Within such tumour context, multiple molecules can contribute to CAFs activation, such as FGF, PDGF, reactive oxygen species (ROS) receptor tyrosine kinase (RTK) TGFβ, TNF [87]. Once activated, CAFs are able to promote tumour growth and cancer invasion. They also interfere with T cell function through the release of exosomes (CAFexo).

**Figure 2:**
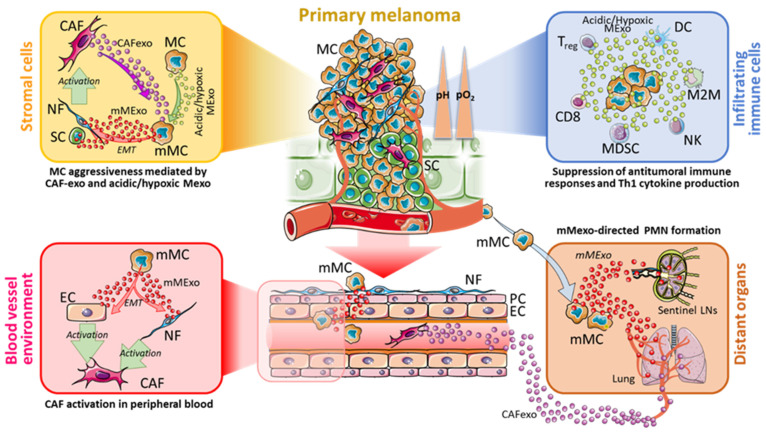
Model of Exosome-based bi-directional crosstalk between CAFs, immune cells and Melanoma. During the development of the primary tumour, CAFs located in the TME release CAFexo which can increase the aggressiveness of MCs. In parallel, MCs in the acidic and hypoxic region (yellow box) can also release acidic and hypoxic Mexo, thus coordinating the generation of mMCs in the acidic and hypoxic regions of melanoma microenvironment, in part via EMT. These mMCs will produce mMexo which, through EMT, do elicit the activation of SCs and NFs into CAFs. In addition, mMCs will migrate into blood vessels and, by releasing mMexo, will contribute to the generation of PMN in distant organs (i.e., lung and LNs), which in turn become educated to receive the migrating mMCs (brown box). In the blood vessel environment (red box) mMCs and CAFs induce ECs and NFs to be activated into CAFs, in part by an EMT-dependent manner. CAFs will release CAFexo which participate in the PMN formation in distant organs, in coordination with mMexo. The acidic Mexo released by MCs will also reach the infiltrating immune cells (light blue box), leading to the activation of MDSCs, M2M and Tregs, and suppression of DCs and NKs. The final effect of such CAF-immune cells-melanoma crosstalk results in the generation of PMNs in distant organs, and the suppression of the anti-tumoural immune responses. CAFexo, CAF-derived exosomes; Mexo, melanoma-derived exosomes; mMexo, metastatic melanoma-derived exosomes; LNs, lymph nodes; NF, normal fibroblast; MC, melanoma cell; mMC, metastatic melanoma cell; EMT, Epithelial-to-mesenchymal transition; SC, skin cell; PC, pericyte; EC, endothelial cells; DC, dendritic cell; M2M, M2-differentiated macrophage; NK, natural killer cell, Treg, T regulatory lymphocyte; CD8, CD8+ T lymphocyte; MDSC, myeloid-derived suppressor cell; PMN, pre-metastatic niche.

Recent studies reported a role of Mexo in the switch from normal fibroblast (NF) to CAF, in turn releasing CAFexo [89] that are able to affect the phenotype of melanoma cells into the TME (Figure 2). Recent findings evidenced that CAFs and melanoma cells can establish an effective bi-directional crosstalk [90], which is likely mediated by their released Exo into the melanoma microenvironment. The main purpose of this mutual crosstalk is to sustain positive feedback between these two cell types that will shape the fate of the skin cancer.

Another cellular component of TME is represented by endothelial cells. In an elegant work performed by using microfluidic devices, Yeon and co-workers demonstrated a relevant role for Mexo in inducing transformation of endothelial cells into CAFs. In addition, they also evidenced that CAFs can migrate towards endothelial-generated vessels (resembling blood circulation) and can interact with endothelial cells to allow their transformation into CAFs via induction of the EMT (Figure 2) [91]. Studies on the effect of acidity on CAFs and CAFexo release are still under investigation. Herein we hypothesize that acidic environment may influence the tumour progression via a complex bidirectional exosome-based crosstalk between CAFs and melanoma cells (Figure 2). In this regard, Izar and co-workers have defined some experimental evidence to support such melanoma-CAF bidirectional network [89,90].

Endothelial cells are also present in blood vessel within TME, [92]. We can hypothesize that TME-resident CAFs can induce EMT to these endothelial cells not only via direct CAF interaction but also via their CAFexo [91]. Therefore, these transformed endothelial cells become CAFs that in turn release Exo. These CAFexo enter into blood circulation and also affect PMN (Figure 2).

Acidity and hypoxia are also able to alter the microenvironment at distant sites to establish a supportive environment called pre-metastatic niche (PMN) that favours the seed for tumour cells and the onset of metastases [93]. In fact, during the early stage of metastasis in several tumours, hypoxia regulates PMN formation by inducing several members of LOX family, including LOX, LOX-like (LOXL) 2 and LOXL4, and by providing cytokines and growth factors recruiting CD11b+ Ly6CmedLy6G+ myeloid cells [94]. Moreover, acidity and hypoxia are also capable of augmenting the secretion of Mexo, which may be associated with PMN formation.

Mexo are able to enter the lymphatic vessels and their pre-conditioning of lymph nodes seems to be an essential prerequisite for the formation of PMN (Figure 2). In support of this, in vivo experiments demonstrated that Mexo influenced the bio-distribution of free melanoma cells within a sentinel lymph node and induced an upregulation of genes involved in cell recruitment, ECM remodelling and vascular proliferation factors, such as tumour necrosis factor (TNF)-α, VEGF, HIF-1 and a urokinase plasminogen activator [54,93]. Mexo secreted by highly metastatic melanoma cells are able to recruit bone marrow progenitor cells toward a pro-vasculogenic phenotype in the metastatic niche, mainly through MET signalling [95,96,97]. Therefore, we can hypothesize that Mexo initially promote angiogenesis to sustain tumour, then migrate to secondary sites to initiate PMN, to finally promote invasion and metastatic colonization to distant sites [98] (Figure 2). Similar roles could be hypothesized also for acidic and hypoxic Mexo.

The preparation of a favourable microenvironment for future metastatic sites can be achieved through docking molecules such as integrins (ITGs) that are expressed on the membrane of tumour exosomes and specifically address Mexo to target organs (organotropism). Multiple studies have shown an altered expression of ITGs in malignant melanoma compared with benign nevi [99,100], and a role in tumour angiogenesis, cell migration, proliferation, and metastasis [101]. Among them, α6β4 and α6β1 ITGs were found expressed on Mexo and, thorough a mechanism based on Src phosphorylation and pro-inflammatory S100 gene expression, they prepared the soil for circulating tumour cells to seed and develop in a target organ, i.e., lung (Figure 2). On the other hand, αvβ5 ITG was linked to liver metastasis [102]. The targeting of α6β4 and αvβ5 resulted in decreased lung and liver metastasis, respectively, thus enforcing their role in metastasis formation [102]. However, how acidity and hypoxia can influence the PMN preparation by ITGs is unknown. What is known is that acidic pHe is able to induce an opening of the ITG dimers α5β1 and αvβ3 headpiece resulting in the activation of ITGs and maturation of focal adhesions, temporal activation of Rho GTPases and microfilament reorganization [103,104]. It is conceivable that such activation may occur also in ITGs at exosomal surface level.

Mexo have also been studied as possible mediator of osteotropism. Although bone metastases are rare in melanoma patients, their onset severely worsens both prognosis and quality of life. A recent work highlights the role of Mexo in reprogramming the innate osteotropism of melanoma cells by up-regulating CXCR7 on cell surface [105].

Altogether, given the high release of Exo triggered by acidity and hypoxia, further studies will be necessary to understand their role in metastasis. The block of Mexo dissemination could represent a new approach to prevent the establishment of cancer cells at secondary organs [106].

## 4. Immune System Modulation under Acidosis and Hypoxia: Role of Mexo

Within TME, acidity and hypoxia impair innate and adaptive antitumor responses. Under hypoxic conditions, HIF activation is responsible for the stimulation, expansion and recruitment of several cell populations causing the suppression of innate and adaptative anticancer immunity [107]. Even acidosis can accomplish the immune-suppression activity operating on proliferation, cytotoxicity, and activation of Natural Killer (NK) and CD8+ cells through inhibition of TH1-type cytokine secretion and lower expression of T-cell receptors [108].

In general, tumour exo can mediate immune suppression both by acting directly through the delivery of apoptosis-inducing mechanisms towards activated immune cells [109], or indirectly through the induction of Regulatory T cell (Treg) differentiation and the expansion of myeloid-derived suppressor cell (MDSC) [110,111,112].

In particular, Mexo were described to encourage the conversion of conventional CD4+CD25– T cells to CD4+ CD25hiFOXP3+ Tregs [110] in a TGF-β1-dependent manner and promote Treg multiplication in vitro [113]. Hence, it follows that the amount of circulating CD4+CD25hiFOXP3+ Tregs is often elevated in cancer patients [114]. Mexo isolated from tumor cell supernatants or obtained from cancer patient plasma [112] contain inhibitory cytokines such as IL-10, TGF-β1 and prostaglandin E2 (PGE2), death receptor ligands such as Fas Ligand or TRAIL, enzymes involved in the adenosine pathway such CD39 and CD73, checkpoint receptor ligands such as PD-L1 [112,115]. Mexo exposed to neutralizing antibody anti TGF-β1 or IL-10 become unable to expand Tregs. Whiteside and co-Workers affirm that once exposed Treg with tumour Exo, these immunosuppressive cells show enhanced suppressive functions and exhibited a greater expression of IL-10, TGF-β, Fas Ligand, CTLA4, granzyme B (GrB) and perforin. In addition, Treg that proliferated in response to tumour exo become totally resistant to Mexo-induced apoptosis [112,113].

However, Mexo interact with immune cells [116] through receptor-mediated internalization [116]. Mexo carry several inhibitory ligands which bind to complementary receptor on immune cells, thus activating negative feedback signals [117]. The principal negatively regulated receptors on immune cells by Mexo are the T cell receptor (TCR) and the IL-2 receptor (IL-2R) [112,118,119]. Nevertheless, the immunostimulatory or immunoinhibitory roles of Mexo might be highly variable, depending on the type of cargo and the functional status of immune cells in TME [112]. In immune cells, exosome-derived miRNAs regulate cell development, cell differentiation and the production of inflammatory mediators. The miRNAs contained in Mexo can play a pivotal role in the regulation/suppression of tumour immune responses [120]. In support of this hypothesis, Vignard and colleagues have elegantly proved that Mexo are able to inhibit the activity of CD8+ T cells through miRNAs (miR-3187-3p, miR-498, miR-122, miR-149, miR-181a/b), that in turn induce a decreased TCR signalling and TNFα secretion [70]. Based on these results such miRNAs could be mediators of tumour evasion and a valuable therapeutic target.

Mexo can regulate the physiology, differentiation, and functions of myeloid cells (monocytes, neutrophils, dendritic cells, macrophages, etc.) [121]. In murine models, Chalmin et al. observed that Mexo are able to mediate an interplay between tumor cells and MDSCs. This interaction that determines the suppressive activity of the MDSC occurs through heat shock protein 72 (Hsp72) triggered by Stat3 activation [122]. Moreover, B16-derived Exo have the ability to induce MDSCs activation in a Toll-like Receptor 2 (TLR2)-dependent manner [123]. Notably, the expression of Hsp72 and TLR-2 is implemented in hypoxic condition, suggesting that hypoxic Mexo can encourage MDSCs functions [124]. Furthermore, also PMRT5 found upregulated in hypoxic Mexo [62], may have a role in the modulation of immune system to promote tumour progression. Indeed, PMRT5 was described to contribute to the germinal centre formation and affinity maturation through BCL6 [125] (Table 1). The germinal centre reaction is important for the generation of humoral immunity and BCL6 is believed to drive the pathogenesis of most B-cell lymphomas. Moreover, PMRT5 was found to be implicated in melanoma response to antitumor immunity. Specifically, this molecule methylates IFI204 and controls NLRC5 expression, which respectively regulates immune cell infiltration and activation, as well as MHCI antigen presentation for tumour cell recognition [126] (Table 1).

Interestingly, it has been recently demonstrated that in hypoxic TME, Mexo are able to influence macrophage recruitment and promote M2-like polarization both in vitro and in vivo [127]. Furthermore, hypoxic Mexo suppress the insulin-Akt-mTOR signalling pathway, through the release of let-7a miRNA, finally enhancing the oxidative phosphorylation in bone marrow-derived macrophages [127]. Therefore, we can hypothesize that Mexo secreted in hypoxic and acidic TME can participate in the modulation of immune system in order to support tumour immune escape (Figure 2, light blue box). Indeed, various proteins that were upregulated in acidic Mexo [66] may have a role in the suppression of the anti-tumour immune response [128] (Table 1). Specifically, GRP78 and HRAS can both contribute to tumour immune escape. GRP78 plays an important role in regulating the activity of TGF-β that acts as an immunosuppressor [129]. Moreover, HRAS is able to regulate the chemotaxis of some immune cells at the tumour stroma [130]. Jolly and co-Workers hypothesized that RAS activation, together with loss of PTEN, induces in the TME the recruitment of immune cells that, in turn, directly promotes tumour metastasis and immune escape [130]. The infiltration of immune cells is enhanced also by GRP94, that in turn interacts with TGF-β and SMAD2 and activates the TGF-β signalling pathway leading to Treg infiltration [131].

Moreover, Mexo can also internalize miR-181d-5p and miR-21-3p (Figure 1), two miRNAs involved in M2 polarization of macrophages [124]. Of note, these miRNAs are found to target HRAS, HYUO1 and GSN, three factors found in acidic Mexo (Figure 1). In this view, it can be hypothesized that miR-181d5p and miR-21-3p are involved in the suppression of infiltrating immune cells in primary melanoma microenvironment by acidic Mexo. Altogether these studies provide evidence that the acidic and hypoxic Mexo molecular content has the potential to impair the anti-tumour immune response (Figure 2). Further studies are necessary to address such hypotheses.

## 5. The Use of Exo in Melanoma Diagnosis, Prognosis and Response to Treatment: Promising Studies

Tumour Exo abundantly present in the body fluids of patients represent a new and powerful analyte in liquid biopsy [132], due to their specific and stage-related markers that monitor and reflect the onset, progression, and prognosis of several type of tumours. However, it must be kept in mind that is still difficult to separate the exosomal fraction from the bulky small vesicles population of different origin that circulate in the blood. As a consequence, the absence of a standardized and optimized procedure and/or the use of different methods of small EVs isolation may hinder comparison between studies [18], thus representing a barrier to the introduction of Exo into clinical use.

Despite these technical limits, however several studies pointed out a potential diagnostic role of Exo markers in melanoma [133]. In fact, several proteins were described in in vitro models and later confirmed on patients’ plasma, such as HSP70, cav-1, TRP-2, Mel-CAM, Mart-1, PMEL, CSPG4, VLA-4, MET, MIA, S100B and PD-L1 (reviewed in [133]). Besides these, several studies highlighted the role of some miRNAs as potential melanoma-derived EVs markers. Furthermore, in this case studies based on patient serum evidenced mir-191, mir-let-7a [59], miR-195, miR-494, miR-665, [55], miR-106b, miR-532-5p [134] as potential genomic markers candidates.

A biochemical approach based on the use of a monoclonal antibody specific for the chondroitin sulfate proteoglycan 4 (CSPG4) epitope, allowed the specific capture and separation of Mexo from Exo derived from normal cells, usually coexisting in patients’ serum [135]. This led to demonstrate that a ratio of tumour over non tumour Exo increased with the disease stage, and that tumour Exo carried an abundance of immunosuppressive proteins and inhibited functions of human primary immune cells [136]. However, CSPG4 is expressed on several cell types [137], therefore its specificity for melanoma remains a question.

In the meantime, to encompass the EVs heterogeneity, some researchers suggested the study of combined genetic markers, which could have also a prognostic value. By a comparison of Exo isolated from melanoma and healthy individuals, Tengda et al. showed that only miRNA-532-5p and miRNA-106b significantly differed and were able to identify with 92% and 88% sensitivity, respectively, the melanoma patients [134]. In addition, higher levels of miR-532-5p and miR-106b were detectable in melanoma patients with stage III–IV disease, as compared to patients with stage I–II disease. In line with this, a recent study evidenced that miR-106b-5p promoted EMT, migration, invasion and adhesion of melanocyte and melanoma metastasis [138].

Another important study reported a melanoma-specific EVs signature, stating the relevance of TRP-2, VLA-4, HSP70, HSP-90 and MET in EVs from plasma of stage IV melanoma patients [97]. Importantly, the co-expression of TYRP2 and MET in Exo, as well as an increased amount of protein per Exo, predicted disease progression in subjects with stage III melanoma. However, GRP94/HSP90 may also have an anti-tumour activity. Indeed, it participates in peptide generation and immune system modulation by targeting proteasome and MHC molecules and activating lymphocytes response [139]. Moreover, GRP94 on Exo surface was further proved to elicit a NK-mediated anti-tumour response, thus representing a potential tumour vaccine [140]. In fact, tumour-derived GRP94 peptide complex is able to activate a potent and cancer cell-specific CD4+ T cell response, and therefore it plays an important role in the promotion of tumour antigen presentation. The ability of this HSP to activate T cell response was investigated as a possible immunotherapy approach for gastric cancer [141].

Several studies indicated ITGs as valuable candidates for anti-metastatic therapy. As previously described, downregulated exosomal α6β4 and αvβ5 ITGs (responsible of PMN formation), lead to a decreased lung liver metastases in an in vivo model [102].. In line with this, several ITG inhibitors, belonging to antibody, peptide or small molecules-based drugs were tested on metastatic melanoma in preclinical studies and clinical trials [101]. Unfortunately, none of them showed efficacy toward metastatic melanoma, being various ITGs responsible for organ-specific metastasis. Therefore, further studies based on an in vivo integrated approach should be developed to understand if the inhibition of ITGs effectively blocks the tumour Exo dissemination and prevents the diffusion of cancer cells at secondary organ sites.

As above described, TME acidification was able to infer a stage-specific melanoma pro-invasive feature through the cell secretion of Exo enriched in proteins (HRAS, GANAB, CFL2, HSP90B1, HSP90AB1, GSN, HSPA1L, NRAS, HSPA5, TIMP3, HYOU1), that were statistically related to melanoma patient’s poor prognosis [66]. It is likely that this signature may be found in stage IV melanoma patient serum Exo.

We have summarized studies describing the upregulation of molecules (proteins, miRNAs) in Mexo released in acidic and hypoxic TME and their role in multiple processes of cancer biology (Table 1). Although performed on cell cultures and mouse models, however the role of the listed molecules was described in several tumour types, and therefore enforced their use as biomarkers candidates. Of course, further studies on Exo from serum patients will be necessary to validate such hypothesis.

As for other tumours, Mexo may represent an important source to evaluate the response to either chirurgical or pharmacological treatments. Indeed, circulating EVs in humans are clearly diminished following tumour resection [142], whereas they are increased in response to chemotherapy [143].

In the last years, the pharmacological treatment based on the block of PD-1/PD-L1 physical interaction gave promising results. Tumour cells evade the immune surveillance by up-regulating surface expression of PD-L1, which interacts with PD-1 on T cells and inhibits T cell killing of tumour cells [144,145]. This opened the way to the development and clinical use of anti-PD1 inhibitor (pembrolizumab) that positively impacted on melanoma patients’ survival [145,146,147]. However, the positive response rate in melanoma patients is still low [146,148] and much effort has to be made in the research community to better understand the PD-L1-mediated immune evasion mechanism. In this regard, a recent study described the EVs-based mechanism of PD-L1 mediated immune evasion [149]. Here, Chen and co-Workers showed that metastatic melanoma patients display a higher level of PD-L1 on the circulating Exo than healthy donors, and purified PD-L1-Exo were able to physically interact and inhibit CD8+ T cells. In addition, Exo derived from melanoma cells treated with IFN-γ exhibited a higher level of binding to CD8+ T cells. Interestingly, this study unveiled for the first time the importance of circulating exosomal PD-L1 as a factor predicting patients’ response and treatment efficacy. Indeed, during anti-PD1 treatment an increase in patients’ exosomal PD-L1 can reflect a successful anti-tumour immune response. Conversely, non-responders to therapy displayed no marked increase in exosomal PD-L1. Therefore, circulating exosomal PD-L1 can be a predictor for the clinical outcomes of anti-PD-1 therapy.

## 6. Conclusions and Future Perspectives

Despite the recent advancements made in the treatment of melanoma, this disease still accounts for significant mortality and an early detection may represent a significant clinical challenge.

EVs, containing a large portion of sEVs/Exo, have been detected in melanoma patients and have been demonstrated to play a prominent role in the disease progression. As a consequence, the interception of such vesicles in patient serum can represent a powerful liquid biopsy option.

A plethora of studies conducted on cell lines indicated a specific stage related signature of Mexo, thus unfolding molecules related to various step of melanoma advancement that could represent promising biomarkers. To circumvent the biological limits displayed by the homogeneous cell line population, we analyzed studies about the melanoma development in its systemic context, mainly represented by acidic and hypoxic microenvironment and by the complex interactions of tumour cell with stromal and immune cells. Acidity and hypoxia are crucial factors in the progress of melanoma, since they can affect the tumour apoptosis, survival, glucose metabolism, angiogenesis, and even response to therapy. Moreover, acidity and hypoxia can orchestrate the multifaceted interplay between tumour, stromal and immune cells, either directly or indirectly through the sEVs/Exo intercellular shuttle, in turn interfering with the cellular functions, and may also participate in the target organ metastases onset through the formation of PMN. Here we focused our analysis to the studies on the characteristics and roles of Mexo in acidic and hypoxic conditions, to disclose potential biomarkers suitable for further clinical use.

Acidity and hypoxia are able to increase the number of released Mexo with a modified molecular content. The new signature is characterized by proteins and miRNAs that sustain tumour progression by inhibition of apoptosis and by promotion of vascularization, cell proliferation, invasion, and migration. Moreover, this signature enhances EMT, induces resistance against chemotherapy and regulates immune response thus contributing to tumour immune escape (Table 1). These combined properties may indicate such exosomal molecules as promising prognostic biomarkers. Of course, a further validation will be necessary in large clinical trials prior to their use as a standard liquid biopsy for monitoring melanoma disease advancement.

To date, although the field of research on Exo is rapidly growing, several obstacles impede their clinical use. One of them is the lack of an elective procedure to separate Exo from other sEVs that are abundantly released by tumour cells, since the available technical procedures do allow the distinction based on size and density, regardless on endosomal or plasma membrane origin of EVs. Further efforts are therefore needed to develop new protocols and techniques that would ensure the isolation of a pure Exo fraction from the bulky vesicular population. Despite these considerations, the study of Exo in general and particularly of acidic and hypoxic Mexo holds promise for the understanding of the melanoma biology, as well as for the identification of circulating biomarkers, and/or new molecular targets exploitable for the development of novel diagnostic, prognostic, and therapeutic programs.

## Figures and Tables

**Table 1:** Molecules upregulated in acidic and hypoxic Mexo. List of molecules upregulated in exosomes secreted in hypoxic (blue) and acidic (orange) TME. Targets and functional effects on tumour progression are described in several tumour types as reported in References. N.D., not detected targets, * Refer to Supplementary Data S1 for the specific reference list.

Molecule	Targets	Functional Effects	Bibliography *
miR-494	BIM; PTEN	Modulates cell proliferation; promotes cell migration and invasion; enhances angiogenesis and tumour growth under hypoxic conditions.	[1,2,3]
miR-1290	SOCS4; LHX6; CCNG2; IKK1; NKD1; INPP4B	Promotes cell proliferation, tumour growth, metastatization and EMT; supresses apoptosis and increases resistance to chemo-radiation	[4,5,6,7,8,9,10]
AKR7A2	Succinic semialdehyde	Supports gamma-Hydroxybutyrate production	[11]
DDX39B	Pre-ribosomal RNA; BRCA1 mRNA	Enhances cell proliferation and increases cell chemotherapy resistance	[12,13]
EIF3C	PI3K/Akt/NF-κB; THBS1, RAP1A, CYR61, ADAMTS1, TUFT1, CFL2, EPHA and EPHB; WNT5B, DHH, SMO, RAC2, PCSK1N and INMBA	Promotes cell proliferation, survival, migration and invasiveness	[14,15,16]
	N.D.	Impairs the sensitivity to EGFR-TKI (Erlotinib) by enhancing the autophagic activity	[17]
FARSA	Activated by FARSA-AS1	Promotes tumour growth and metastasis	[18]
circFARSA	miR-330-5p/LASP1	Enhance proliferation, migration, and invasion	[19]
PMRT5	H3R8, H4R3 and RBL2; CUL4AB promoters; PDCD4; eIF4E; E2F1; HIF-1	Promotes cancer cell proliferation and inhibits apoptosis	[20,21,22,23,24,25]
	MDM4	Supports drug resistance to palbociclib	[26]
	IFI16 and NLRC5	Impairs antitumor immune response	[27]
	BCL6	Regulates the germinal centre reaction	[28]
VARS	TCTP-EF1A2	Promotes tumorigenesis and tumour progression	[29,30]
hnRNP L	miR-574-3p; AXL; SRSF3; p53, Bcl-2, caspases-3, -6, -9 and MAPK; SPRY4-IT1	Promotes cell proliferation and inhibits apoptosis, enhances tumorigenesis and the tumour metastatic potential	[31,32,33,34,35,36]
hnRNP K	MAP 1B-LC1; SRSF1; DUSP1; EZH2-SOX2; CCND1, G0S2, XAF1 and ERCC4; MMP12 and ß-catenin; XIAP; GSN mRNA; MMP-2	Promotes EMT, cell proliferation, migration and invasion; role in chemoresistance	[37,38,39,40,41,42,43,44]
	PTGS2, CCK3, RAS, ERK and MMP-3.	Promotes tumour metastasis regulating extracellular matrix, cell motility, and angiogenesis pathways	[45]
RAN	Importin-β; RhoA; Caspase-3; AR; Met and c-Met receptor; PI3 kinase; Txl-2b; AR and CXCR4; Cyclin A, Cyclin D1, Cyclin E, CDK2, CDK4, phospho-Rb and Survivin; PI3K/Akt/mTORC1 and Ras/MEK/ERK; FGF2; Aurora Kinase A; miR-21	Enhances tumorigenesis; inhibits apoptosis; promotes cell proliferation, migration and invasion; promotes gefitinib resistance	[46,47,48,49,50,51,52,53,54,55,56]
NRAS and HRAS	BRAF, PI3K, PLC/PKC, RAL	Promote proliferation, survival and cell growth, and enhances cell migration	[57]
	SDF-1, I-TAC, CCL9/10, and MCP5	Induces the tumour immune escape	[58]
HSPA1L	IGF1Rβ and β-Catenin	Enhances EMT and Cancer Stem Cell-like properties	[59]
	HIF-1α/GP78	Promotes the cellular prion protein (PrPC) accumulation and tumorigenicity	[60]
HSPA5 (GRP78)	VEGFR-2; Kringle 5	Promotes vascularization	[61,62]
	TFEB, CSTD, CTSL, and LAMP1	Supports cell protection against ER stress and ROS damage; regulates lysosomal activity	[63]
	ATF4-DDIT4-mTORC1	Induces pro-survival autophagy	[64]
	Cripto; Proteinase inhibitor α2-macroglobulin (α2M *); FAK; PRMT7; PI3K/Akt/Mdm2	Activates EMT and promotes cell proliferation, migration and invasion	[65,66,67,68,69]
	Raf-1; Caspase-7; PERK; Wnt	Inhibits apoptosis and enhances cell survival	[70,71,72,73]
	CD5L; CHOP, Bcl-2 and Bax; GPX4; ERK/AKT and BOK/NOXA	Involved in chemoresistance	[74,75,76,77]
	IL-10, TGF-β and IDO; PD-L1; LAP/TGF-beta	Supports tumour immune escape; cell protection from CTL-mediated lysis	[78,79,80]
HSP90B1 (GRP94 and GP96)	Twist1	Promotes vasculogenic mimicry	[81]
	CCT8/c-Jun; AKT and eNOS; TGF-β1; Ack1; RAC1, VAMP2, LAMP1, SYNE2, integrin α2/αL; Wnt/β-catenin	Contributes to tumorigenesis and promotes cell migration, invasion and metastasis	[82,83,84,85,86,87]
	Mdm2 E3 ligase	Inhibits apoptosis and decreases p53 levels	[88]
	Proteasome and MHC molecules	Participates in peptide generation and modulates immune system	[89]
	TGF-β and SMAD2	Induces Treg infiltration by promoting the TGF-β signalling pathway.	[90]
	TNF-α, IL-10, IL-12 p70 and IFN-γ	Promotes T cell response, enhances DC antigen presentation and induces cytokine secretion	[91]
HSP90AB1	VEGFR	Promotes endothelial tumor angiogenesis and accelerates neovascularization	[92]
	Bcl-2	Inhibits cell apoptosis and increases the caspase activation	[93]
	Fibronectin	Induces fibronectin exocytosis and formation of extracellular matrix	[94]
	ERBB2 and CDK4; LRP5	Promotes cell proliferation, invasion and migration, tolerance to chemotherapeutic drugs and in vivo metastasis	[95,96]
CFL2	miR-3189-3p; miR-369-3p; miR-1299	Enhances cell proliferation and migration; impairs tumour cell apoptosis	[97,98,99]
GSN	Nm23-H1; MCL-1, MMP-2 and MMP-9; p-AKT and p-P38	Promotes cell growth, migration and invasion and in vivo tumour growth	[100,101,102]
	XIAP, FLIP, Akt and AIF; PSME2, PTK2B, FOS, JUN, ITGB1, MAP2K7, MAP3K4, MAP3K12, Rac1 and RRM2B	Inhibits apoptosis; supports the response to cisplatin	[103,104]
TIMP3	Tnfr1	Supports early stage of tumorigenesis	[105]
GANAB	Wnt/β-catenin	Promotes proliferation and suppresses apoptosis	[106,107]
HYOU1	LDHB mRNA; IFN-type I; CHOP; VEGF; PI3K/AKT; MMP-2	Promotes proliferation, migration, invasion of cancer cells and inhibits apoptosis	[108,109,110,111,112,113]
Sphingo-mielin	KRAS	Increases the growth of oncogenic K-Ras-transformed tumours	[114]
	EGFR/ErbB1	Modulates cell motility and focal adhesion clustering	[115]

**Table 2:** Effects of hypoxic miRNAs listed in Figure 1 (red arrowheads) on melanoma progression, metastasis, and therapy resistance. Mt, Metastatic expansion; Pr, Proliferation; Is, Immunosuppression; Dr, Drug resistance; EMT, Epithelial to Mesenchimal Transition; * Refer to Supplementary Materials for the specific reference list.

miRNA Name	Involved Process	Oncomir	Effects	References *
hsa-let-7f-5p	Mt	No	Interferes with cell anchorage and promotes cell cycle process during metastatic expansion	[116]
hsa-mir-10b-5p	Mt	Yes	Promotes progression and metastasis through by donwregulation of ITCH in Wnt/beta-Catenin pathway	[117]
hsa-mir-125a-3p	Mt, Dr	No	Promotes melanoma progression and metastasis via Lin28B protein; Promotes melanoma resistance to the BRAF inhibitor Vemurafenib via suppression of apoptotic pathways	[118,119]
hsa-mir-150-5p	Mt	No	Key regulator of proliferation, invasion and glycogenesis in malignant melanoma through SIX1	[120]
hsa-mir-152-3p	Mt	No	Promotes malignant melanoma progression by binding to the lncRNA HOTAIR	[121]
hsa-mir-155-5p	Pr	No	Can increase melanoma progression by modulation of the SKI factor	[122]
hsa-mir-181b-3p hsa-mir-181b-5p hsa-mir-181d-5p	Pr	No	Elicit melanoma cell cycle by targeting the CTDSPL protein	[123]
hsa-mir-188-3p	Mt	No	Sustains melanoma progression via Mesenchimal Stem Cell reprogramming	[124]
hsa-mir-191-3p hsa-mir-191-5p	Pr	No	Associated with poor survival in melanoma patients	[125]
hsa-mir-196a-5p	Pr	No	Aberrantly expressed in melanoma by disregulation of HOX-C8 expression	[126]
hsa-mir-199a-3p	Mt	Yes	Promotion of melanoma metastasis and angiogenesis by targeting the ApoE lipoprotein	[127]
hsa-mir-200a-3p	Mt, Dr	No	Reduced response to CDK4/6 inhibitor in highly proliferative metastatic melanoma via diminished expression of CDK6	[128]
hsa-mir-200b-3p	Pr	No	Activates melanoma invasiveness, progression and EMT via the NEAT1/SMAD2 axis	[129]
hsa-mir-203a-3p	Mt	Yes	Promotion of stemness, increased BRAF expression and augmented tumorigenesis in melanoma cell lines and in vivo	[130]
hsa-mir-210-3p	Is	No	Promotes melanoma progression by hypoxia-induced immunosuppression in oxygen-deprived regions of the melanoma microenvironment favoring the evolution of cancer stem cells and the resistance to drug therapy	[131]
hsa-mir-21-3p	Mt	Yes	Promotes melanoma cell invasiveness by decreasing the expression of the tissue Metalloproteinase 3 inhibitors in vivo Enhances melanoma invasion and metastasis by promoting the insurgence of NRAS and BRAF mutation and by increasing the expression of L1CAM	[132,133]
hsa-mir-21-5p	Pr	Yes	Targets CDKN2C and activates melanoma cell progression and G1/S transition	[134]
hsa-mir-224-5p	Mt	Yes	Induction of EMT by TXNIP downregulation	[135]
hsa-mir-24-3p	Mt, Dr	No	Confers resistance to Vemurafenib through the occurrence of BRAF mutations in melanoma	[136]
hsa-mir-26b-5p	Is	No	Elicits melanoma progression by favoring the HLA class I-mediated immune escape	[137]
hsa-mir-30a-5p	Dr	No	Confers resistance to Cisplatin by targeting the IGF1R gene	[138]
hsa-mir-30d-3p hsa-mir-30d-5p	Mt, Is	Yes	Enhances melanoma cell invasiveness and immunosuppression (via increasing Treg cells) during metastatic expansion by modulation of GalNAc transferases	[139]
hsa-mir-373-3p	Mt, Is	Yes	Decreases immunovisibility to melanoma thus increasing tumour dissemination; Promotion of melanoma cell invasiveness by SIK1 targeting	[140,141]
hsa-mir-93	Mt	No	Found upmodulated in melanoma metastases	[142]

## Data Availability

Data generated in Figure 1 were obtained by interrogating the miRNet public database on the Gene list panel tab, available at https://www.mirnet.ca/, accessed on 30 March 2021. In this tab, input data is represented by the following parameters: Organism: H.Sapiens (human); ID Type: Official Gene symbol; Tissue: exosomes (1250); Targeted by: miRNA; Gene list: HRAS, GANAB, CFL2, HSP90B1, HSP90AB1, GSN, HSPA1L, NRAS, HSPA5, TIMP3, HYOU1.

## Supplementary Materials

The following are available online at https://www.mdpi.com/article/10.3390/cells10123311/s1, Reference list for Table 1 and Table 2.
