# Supplementary material for: Acidic and Hypoxic Microenvironment in Melanoma: Impact of Tumour Exosomes on Disease Progression
Source: Cells. 2021 Nov 25;10(12):3311. doi: 10.3390/cells10123311 (PMC8699343; doi:10.3390/cells10123311)
Supplement: Supplementary file 1 [file cells-10-03311-s001.zip › cells-1459437-supplementary.pdf]

Supplementary references for Table 1 and Table 2

No. Reference details

1. Sun, H.B.; Chen, X.; Ji, H.; Wu, T.; Lu, H.W.; Zhang, Y.; Li, H.; Li, Y.M. miR-494 is an independent prognostic factor and promotes cell migration and invasion in colorectal cancer by directly targeting PTEN. Int J Oncol 2014, 45, 2486-2494, doi:10.3892/ijo.2014.2665.

2. Liu, Y.; Lai, L.; Chen, Q.; Song, Y.; Xu, S.; Ma, F.; Wang, X.; Wang, J.; Yu, H.; Cao, X.; et al. MicroRNA-494 is required for the accumulation and functions of tumor-expanded myeloid-derived suppressor cells via targeting of PTEN. J Immunol 2012, 188, 5500-5510, doi:10.4049/jimmunol.1103505.

3. Mao, G.; Liu, Y.; Fang, X.; Fang, L.; Lin, L.; Liu, X.; Wang, N. Tumor-derived microRNA-494 promotes angiogenesis in non-small cell lung cancer. Angiogenesis 2015, 18, 373-382, doi:10.1007/s10456-015-9474-5.

4. Xiao, X.; Yang, D.; Gong, X.; Mo, D.; Pan, S.; Xu, J. miR-1290 promotes lung adenocarcinoma cell proliferation and invasion by targeting SOCS4. Oncotarget 2018, 9, 11977-11988, doi:10.18632/oncotarget.24046.

5. Khalighfard, S.; Kalhori, M.R.; Haddad, P.; Khori, V.; Alizadeh, A.M. Enhancement of resistance to chemo-radiation by hsa-miR-1290 expression in glioblastoma cells. Eur J Pharmacol 2020, 880, 173144, doi:10.1016/j.ejphar.2020.173144.

6. Yan, L.; Cai, K.; Sun, K.; Gui, J.; Liang, J. MiR-1290 promotes proliferation, migration, and invasion of glioma cells by targeting LHX6. J Cell Physiol 2018, 233, 6621-6629, doi:10.1002/jcp.26381.

7. Qin, W.J.; Wang, W.P.; Wang, X.B.; Zhang, X.T.; Du, J.D. MiR-1290 targets CCNG2 to promote the metastasis of oral squamous cell carcinoma. Eur Rev Med Pharmacol Sci 2019, 23, 10332-10342, doi:10.26355/eurrev_201912_19671.

8. Ta, N.; Huang, X.; Zheng, K.; Zhang, Y.; Gao, Y.; Deng, L.; Zhang, B.; Jiang, H.; Zheng, J. miRNA-1290 Promotes Aggressiveness in Pancreatic Ductal Adenocarcinoma by Targeting IKK1. Cell Physiol Biochem 2018, 51, 711-728, doi:10.1159/000495328.

9. Huang, J.; Shen, M.; Yan, M.; Cui, Y.; Gao, Z.; Meng, X. Exosome-mediated transfer of miR-1290 promotes cell proliferation and invasion in gastric cancer via NKD1. Acta Biochim Biophys Sin (Shanghai) 2019, 51, 900-907, doi:10.1093/abbs/gmz077.

10. Ma, Q.; Wang, Y.; Zhang, H.; Wang, F. miR-1290 Contributes to Colorectal Cancer Cell Proliferation by Targeting INPP4B. Oncol Res 2018, 26, 1167-1174, doi:10.3727/096504017X15051741798389.

11. Lyon, R.C.; Johnston, S.M.; Watson, D.G.; McGarvie, G.; Ellis, E.M. Synthesis and catabolism of gamma-hydroxybutyrate in SH-SY5Y human neuroblastoma cells: role of the aldo-keto reductase AKR7A2. J Biol Chem 2007, 282, 25986-25992, doi:10.1074/jbc.M702465200.

12. Awasthi, S.; Chakrapani, B.; Mahesh, A.; Chavali, P.L.; Chavali, S.; Dhayalan, A. DDX39B promotes translation through regulation of pre-ribosomal RNA levels. RNA Biol 2018, 15, 1157-1166, doi:10.1080/15476286.2018.1517011.

13. Xu, Z.; Li, X.; Li, H.; Nie, C.; Liu, W.; Li, S.; Liu, Z.; Wang, W.; Wang, J. Suppression of DDX39B sensitizes ovarian cancer cells to DNA-damaging chemotherapeutic agents via destabilizing BRCA1 mRNA. Oncogene 2020, 39, 7051-7062, doi:10.1038/s41388-020-01482-x.

14. Hu, J.; Luo, H.; Xu, Y.; Luo, G.; Xu, S.; Zhu, J.; Song, D.; Sun, Z.; Kuang, Y. The Prognostic Significance of. Cancer Invest 2019, 37, 199-208, doi:10.1080/07357907.2019.1618322.

15. Wen, F.; Wu, Z.Y.; Nie, L.; Zhang, Q.Z.; Qin, Y.K.; Zhou, Z.L.; Wu, J.J.; Zhao, X.; Tan, J.; Sawmiller, D.; et al. Eukaryotic initiation factor 3, subunit C silencing inhibits cell proliferation and promotes apoptosis in human ovarian cancer cells. Biosci Rep 2019, 39, doi:10.1042/BSR20191124.

16. Li, T.; Li, S.; Chen, D.; Chen, B.; Yu, T.; Zhao, F.; Wang, Q.; Yao, M.; Huang, S.; Chen, Z.; et al. Transcriptomic analyses of RNA-binding proteins reveal eIF3c promotes cell proliferation in hepatocellular carcinoma. Cancer Sci 2017, 108, 877-885, doi:10.1111/cas.13209.

17. Shintani, T.; Higashisaka, K.; Maeda, M.; Hamada, M.; Tsuji, R.; Kurihara, K.; Kashiwagi, Y.; Sato, A.; Obana, M.; Yamamoto, A.; et al. Eukaryotic translation initiation factor 3 subunit C is associated with acquired resistance to erlotinib in non-small cell lung cancer. Oncotarget 2018, 9, 37520-37533, doi:10.18632/oncotarget.26494.

18. Zhou, T.; Wu, L.; Ma, N.; Tang, F.; Yu, Z.; Jiang, Z.; Li, Y.; Zong, Z.; Hu, K. SOX9-activated FARSA-AS1 predetermines cell growth, stemness, and metastasis in colorectal cancer through upregulating FARSA and SOX9. Cell Death Dis 2020, 11, 1071, doi:10.1038/s41419-020-03273-4.

19. Lu, C.; Fu, L.; Qian, X.; Dou, L.; Cang, S. Knockdown of circular RNA circ-FARSA restricts colorectal cancer cell growth through regulation of miR-330-5p/LASP1 axis. Arch Biochem Biophys 2020, 689, 108434, doi:10.1016/j.abb.2020.108434.

20. Wang, L.; Pal, S.; Sif, S. Protein arginine methyltransferase 5 suppresses the transcription of the RB family of tumor suppressors in leukemia and lymphoma cells. Mol Cell Biol 2008, 28, 6262-6277, doi:10.1128/MCB.00923-08.

21. Aggarwal, P.; Vaites, L.P.; Kim, J.K.; Mellert, H.; Gurung, B.; Nakagawa, H.; Herlyn, M.; Hua, X.; Rustgi, A.K.; McMahon, S.B.; et al. Nuclear cyclin D1/CDK4 kinase regulates CUL4 expression and triggers neoplastic growth via activation of the PRMT5 methyltransferase. Cancer Cell 2010, 18, 329-340, doi:10.1016/j.ccr.2010.08.012.

22. Powers, M.A.; Fay, M.M.; Factor, R.E.; Welm, A.L.; Ullman, K.S. Protein arginine methyltransferase 5 accelerates tumor growth by arginine methylation of the tumor suppressor programmed cell death 4. Cancer Res 2011, 71, 5579-5587, doi:10.1158/0008-5472.CAN-11-0458.

23. Scoumanne, A.; Zhang, J.; Chen, X. PRMT5 is required for cell-cycle progression and p53 tumor suppressor function. Nucleic Acids Res 2009, 37, 4965-4976, doi:10.1093/nar/gkp516.

24. Cho, E.C.; Zheng, S.; Munro, S.; Liu, G.; Carr, S.M.; Moehlenbrink, J.; Lu, Y.C.; Stimson, L.; Khan, O.; Konietzny, R.; et al. Arginine methylation controls growth regulation by E2F-1. EMBO J 2012, 31, 1785-1797, doi:10.1038/emboj.2012.17.

25. Lim, J.H.; Choi, Y.J.; Cho, C.H.; Park, J.W. Protein arginine methyltransferase 5 is an essential component of the hypoxia-inducible factor 1 signaling pathway. Biochem Biophys Res Commun 2012, 418, 254-259, doi:10.1016/j.bbrc.2012.01.006.

26. AbuHammad, S.; Cullinane, C.; Martin, C.; Bacolas, Z.; Ward, T.; Chen, H.; Slater, A.; Ardley, K.; Kirby, L.; Chan, K.T.; et al. Regulation of PRMT5-MDM4 axis is critical in the response to CDK4/6 inhibitors in melanoma. Proc Natl Acad Sci U S A 2019, 116, 17990-18000, doi:10.1073/pnas.1901323116.

27. Kim, H.; Feng, Y.; Li, Y.; Tamiya, H.; Tocci, S.; Ronai, Z.A. PRMT5 control of cGAS/STING and NLRC5 pathways defines melanoma response to antitumor immunity. Sci Transl Med 2020, 12, doi:10.1126/scitranslmed.aaz5683.

28. Lu, X.; Fernando, T.M.; Lossos, C.; Yusufova, N.; Liu, F.; Fontán, L.; Durant, M.; Geng, H.; Melnick, J.; Luo, Y.; et al. PRMT5 interacts with the BCL6 oncoprotein and is required for germinal center formation and lymphoma cell survival. Blood 2018, 132, 2026-2039, doi:10.1182/blood-2018-02-831438.

29. Kim, Y.W.; Kwon, C.; Liu, J.L.; Kim, S.H.; Kim, S. Cancer association study of aminoacyl-tRNA synthetase signaling network in glioblastoma. PLoS One 2012, 7, e40960, doi:10.1371/journal.pone.0040960.

30. Kobayashi, D.; Tokuda, T.; Sato, K.; Okanishi, H.; Nagayama, M.; Hirayama-Kurogi, M.; Ohtsuki, S.; Araki, N. Identification of a Specific Translational Machinery via TCTP-EF1A2 Interaction Regulating NF1-associated Tumor Growth by Affinity Purification and Data-independent Mass Spectrometry Acquisition (AP-DIA). Mol Cell Proteomics 2019, 18, 245-262, doi:10.1074/mcp.RA118.001014.

31. Yao, P.; Wu, J.; Lindner, D.; Fox, P.L. Interplay between miR-574-3p and hnRNP L regulates VEGFA mRNA translation and tumorigenesis. Nucleic Acids Res 2017, 45, 7950-7964, doi:10.1093/nar/gkx440.

32. Kawasaki, Y.; Miyamoto, M.; Oda, T.; Matsumura, K.; Negishi, L.; Nakato, R.; Suda, S.; Yokota, N.; Shirahige, K.; Akiyama, T. The novel lncRNA CALIC upregulates AXL to promote colon cancer metastasis. EMBO Rep 2019, 20, e47052, doi:10.15252/embr.201847052.

33. Jia, R.; Zhang, S.; Liu, M.; Zhang, Y.; Liu, Y.; Fan, M.; Guo, J. HnRNP L is important for the expression of oncogene SRSF3 and oncogenic potential of oral squamous cell carcinoma cells. Sci Rep 2016, 6, 35976, doi:10.1038/srep35976.

34. Zhou, X.; Li, Q.; He, J.; Zhong, L.; Shu, F.; Xing, R.; Lv, D.; Lei, B.; Wan, B.; Yang, Y.; et al. HnRNP-L promotes prostate cancer progression by enhancing cell cycling and inhibiting apoptosis. Oncotarget 2017, 8, 19342-19353, doi:10.18632/oncotarget.14258.

35. Lv, D.; Wu, H.; Xing, R.; Shu, F.; Lei, B.; Lei, C.; Zhou, X.; Wan, B.; Yang, Y.; Zhong, L.; et al. HnRNP-L mediates bladder cancer progression by inhibiting apoptotic signaling and enhancing MAPK signaling pathways. Oncotarget 2017, 8, 13586-13599, doi:10.18632/oncotarget.14600.

36. Ma, W.; Chen, X.; Wu, X.; Li, J.; Mei, C.; Jing, W.; Teng, L.; Tu, H.; Jiang, X.; Wang, G.; et al. Long noncoding RNA SPRY4-IT1 promotes proliferation and metastasis of hepatocellular carcinoma via mediating TNF signaling pathway. J Cell Physiol 2020, 235, 7849-7862, doi:10.1002/jcp.29438.

37. Li, L.; Yan, S.; Zhang, H.; Zhang, M.; Huang, G.; Chen, M. Interaction of hnRNP K with MAP 1B-LC1 promotes TGF-β1-mediated epithelial to mesenchymal transition in lung cancer cells. BMC Cancer 2019, 19, 894, doi:10.1186/s12885-019-6119-x.

38. Peng, W.Z.; Liu, J.X.; Li, C.F.; Ma, R.; Jie, J.Z. hnRNPK promotes gastric tumorigenesis through regulating CD44E alternative splicing. Cancer Cell Int 2019, 19, 335, doi:10.1186/s12935-019-1020-x.

39. Peng, W.; Zhang, C.; Peng, J.; Huang, Y.; Peng, C.; Tan, Y.; Ji, D.; Zhang, Y.; Zhang, D.; Tang, J.; et al. Lnc-FAM84B-4 acts as an oncogenic lncRNA by interacting with protein hnRNPK to restrain MAPK phosphatases-DUSP1 expression. Cancer Lett 2020, 494, 94-106, doi:10.1016/j.canlet.2020.08.036.

40. Chen, X.; Gu, P.; Xie, R.; Han, J.; Liu, H.; Wang, B.; Xie, W.; Zhong, G.; Chen, C.; Xie, S.; et al. Heterogeneous nuclear ribonucleoprotein K is associated with poor prognosis and regulates proliferation and apoptosis in bladder cancer. J Cell Mol Med 2017, 21, 1266-1279, doi:10.1111/jcmm.12999.

41. Tsai, H.Y.; Fu, S.L.; Tseng, L.M.; Chiu, J.H.; Lin, C.H. hnRNPK S379 phosphorylation participates in migration regulation of triple negative MDA-MB-231 cells. Sci Rep 2019, 9, 7611, doi:10.1038/s41598-019-44063-z.

42. Xiao, Z.; Ko, H.L.; Goh, E.H.; Wang, B.; Ren, E.C. hnRNP K suppresses apoptosis independent of p53 status by maintaining high levels of endogenous caspase inhibitors. Carcinogenesis 2013, 34, 1458-1467, doi:10.1093/carcin/bgt085.

43. Liu, X.H.; Ma, J.; Feng, J.X.; Feng, Y.; Zhang, Y.F.; Liu, L.X. Regulation and related mechanism of GSN mRNA level by hnRNPK in lung adenocarcinoma cells. Biol Chem 2019, 400, 951-963, doi:10.1515/hsz-2018-0417.

44. Otoshi, T.; Tanaka, T.; Morimoto, K.; Nakatani, T. Cytoplasmic Accumulation of Heterogeneous Nuclear Ribonucleoprotein K Strongly Promotes Tumor Invasion in Renal Cell Carcinoma Cells. PLoS One 2015, 10, e0145769, doi:10.1371/journal.pone.0145769.

45. Gao, R.; Yu, Y.; Inoue, A.; Widodo, N.; Kaul, S.C.; Wadhwa, R. Heterogeneous nuclear ribonucleoprotein K (hnRNP-K) promotes tumor metastasis by induction of genes involved in extracellular matrix, cell movement, and angiogenesis. J Biol Chem 2013, 288, 15046-15056, doi:10.1074/jbc.M113.466136.

46. Dell'Anno, I.; Barbarino, M.; Barone, E.; Giordano, A.; Luzzi, L.; Bottaro, M.; Migliore, L.; Agostini, S.; Melani, A.; Melaiu, O.; et al. and. Int J Mol Sci 2020, 21, doi:10.3390/ijms21144856.

47. Zaoui, K.; Boudhraa, Z.; Khalifé, P.; Carmona, E.; Provencher, D.; Mes-Masson, A.M. Ran promotes membrane targeting and stabilization of RhoA to orchestrate ovarian cancer cell invasion. Nat Commun 2019, 10, 2666, doi:10.1038/s41467-019-10570-w.

48. Yuen, H.F.; Chan, K.K.; Grills, C.; Murray, J.T.; Platt-Higgins, A.; Eldin, O.S.; O'Byrne, K.; Janne, P.; Fennell, D.A.; Johnston, P.G.; et al. Ran is a potential therapeutic target for cancer cells with molecular changes associated with activation of the PI3K/Akt/mTORC1 and Ras/MEK/ERK pathways. Clin Cancer Res 2012, 18, 380-391, doi:10.1158/1078-0432.CCR-11-2035.

49. Wang, F.; Yang, L.; Shi, L.; Li, Q.; Zhang, G.; Wu, J.; Zheng, J.; Jiao, B. Nuclear translocation of fibroblast growth factor-2 (FGF2) is regulated by Karyopherin-β2 and Ran GTPase in human glioblastoma cells. Oncotarget 2015, 6, 21468-21478, doi:10.18632/oncotarget.4097.

50. Caputo, E.; Wang, E.; Valentino, A.; Crispi, S.; De Giorgi, V.; Fico, A.; Ficili, B.; Capone, M.; Anniciello, A.; Cavalcanti, E.; et al. Ran signaling in melanoma: implications for the development of alternative therapeutic strategies. Cancer Lett 2015, 357, 286-296, doi:10.1016/j.canlet.2014.11.033.

51. Zhang, F.; Yang, Z.; Cao, M.; Xu, Y.; Li, J.; Chen, X.; Gao, Z.; Xin, J.; Zhou, S.; Zhou, Z.; et al. MiR-203 suppresses tumor growth and invasion and down-regulates MiR-21 expression through repressing Ran in esophageal cancer. Cancer Lett 2014, 342, 121-129, doi:10.1016/j.canlet.2013.08.037.

52. Barrès, V.; Ouellet, V.; Lafontaine, J.; Tonin, P.N.; Provencher, D.M.; Mes-Masson, A.M. An essential role for Ran GTPase in epithelial ovarian cancer cell survival. Mol Cancer 2010, 9, 272, doi:10.1186/1476-4598-9-272.

53. Deng, Q.; Luo, L.; Quan, Z.; Liu, N.; Du, Z.; Sun, W.; Luo, C.; Wu, X. HepaCAM inhibits cell proliferation and invasion in prostate cancer by suppressing nuclear translocation of the androgen receptor via its cytoplasmic domain. Mol Med Rep 2019, 19, 2115-2124, doi:10.3892/mmr.2019.9841.

54. Yuen, H.F.; Chan, K.K.; Platt-Higgins, A.; Dakir, e.-H.; Matchett, K.B.; Haggag, Y.A.; Jithesh, P.V.; Habib, T.; Faheem, A.; Dean, F.A.; et al. Ran GTPase promotes cancer progression via Met recepto-rmediated downstream signaling. Oncotarget 2016, 7, 75854-75864, doi:10.18632/oncotarget.12420.

55. Zhang, C.; Zhao, X.; Du, W.; Shen, J.; Li, S.; Li, Z.; Wang, Z.; Liu, F. Ran promotes the proliferation and migration ability of head and neck squamous cell carcinoma cells. Pathol Res Pract 2020, 216, 152951, doi:10.1016/j.prp.2020.152951.

56. Kurisetty, V.V.; Johnston, P.G.; Johnston, N.; Erwin, P.; Crowe, P.; Fernig, D.G.; Campbell, F.C.; Anderson, I.P.; Rudland, P.S.; El-Tanani, M.K. RAN GTPase is an effector of the invasive/metastatic phenotype induced by osteopontin. Oncogene 2008, 27, 7139-7149, doi:10.1038/onc.2008.325.

57. Cicenas, J.; Tamosaitis, L.; Kvederaviciute, K.; Tarvydas, R.; Staniute, G.; Kalyan, K.; Meskinyte-Kausiliene, E.; Stankevicius, V.; Valius, M. KRAS, NRAS and BRAF mutations in colorectal cancer and melanoma. Med Oncol 2017, 34, 26, doi:10.1007/s12032-016-0879-9.

58. Jolly, L.A.; Massoll, N.; Franco, A.T. Immune Suppression Mediated by Myeloid and Lymphoid Derived Immune Cells in the Tumor Microenvironment Facilitates Progression of Thyroid Cancers Driven by Hras. J Clin Cell Immunol 2016, 7, doi:10.4172/2155-9899.1000451.

59. Choi, S.I.; Lee, J.H.; Kim, R.K.; Jung, U.; Kahm, Y.J.; Cho, E.W.; Kim, I.G. HSPA1L Enhances Cancer Stem Cell-Like Properties by Activating IGF1Rβ and Regulating β-Catenin Transcription. Int J Mol Sci 2020, 21, doi:10.3390/ijms21186957.

60. Lee, J.H.; Han, Y.S.; Yoon, Y.M.; Yun, C.W.; Yun, S.P.; Kim, S.M.; Kwon, H.Y.; Jeong, D.; Baek, M.J.; Lee, H.J.; et al. Role of HSPA1L as a cellular prion protein stabilizer in tumor progression via HIF-1α/GP78 axis. Oncogene 2017, 36, 6555-6567, doi:10.1038/onc.2017.263.

61. Katanasaka, Y.; Ishii, T.; Asai, T.; Naitou, H.; Maeda, N.; Koizumi, F.; Miyagawa, S.; Ohashi, N.; Oku, N. Cancer antineovascular therapy with liposome drug delivery systems targeted to BiP/GRP78. Int J Cancer 2010, 127, 2685-2698, doi:10.1002/ijc.25276.

62. Davidson, D.J.; Haskell, C.; Majest, S.; Kherzai, A.; Egan, D.A.; Walter, K.A.; Schneider, A.; Gubbins, E.F.; Solomon, L.; Chen, Z.; et al. Kringle 5 of human plasminogen induces apoptosis of endothelial and tumor cells through surface-expressed glucose-regulated protein 78. Cancer Res 2005, 65, 4663-4672, doi:10.1158/0008-5472.CAN-04-3426.

63. Kim, S.Y.; Kim, H.J.; Kim, D.H.; Han, J.H.; Byeon, H.K.; Lee, K.; Kim, C.H. HSPA5 negatively regulates lysosomal activity through ubiquitination of MUL1 in head and neck cancer. Autophagy 2018, 14, 385-403, doi:10.1080/15548627.2017.1414126.

64. Mu, N.; Lei, Y.; Wang, Y.; Duan, Q.; Ma, G.; Liu, X.; Su, L. Inhibition of SIRT1/2 upregulates HSPA5 acetylation and induces pro-survival autophagy via ATF4-DDIT4-mTORC1 axis in human lung cancer cells. Apoptosis 2019, 24, 798-811, doi:10.1007/s10495-019-01559-3.

65. Shani, G.; Fischer, W.H.; Justice, N.J.; Kelber, J.A.; Vale, W.; Gray, P.C. GRP78 and Cripto form a complex at the cell surface and collaborate to inhibit transforming growth factor beta signaling and enhance cell growth. Mol Cell Biol 2008, 28, 666-677, doi:10.1128/MCB.01716-07.

66. Misra, U.K.; Deedwania, R.; Pizzo, S.V. Activation and cross-talk between Akt, NF-kappaB, and unfolded protein response signaling in 1-LN prostate cancer cells consequent to ligation of cell surface-associated GRP78. J Biol Chem 2006, 281, 13694-13707, doi:10.1074/jbc.M511694200.

67. Su, R.; Li, Z.; Li, H.; Song, H.; Bao, C.; Wei, J.; Cheng, L. Grp78 promotes the invasion of hepatocellular carcinoma. BMC Cancer 2010, 10, 20, doi:10.1186/1471-2407-10-20.

68. Cheng, D.; He, Z.; Zheng, L.; Xie, D.; Dong, S.; Zhang, P. contributes to the metastasis phenotype in human non-small-cell lung cancer cells possibly through the interaction with. Onco Targets Ther 2018, 11, 4869-4876, doi:10.2147/OTT.S166412.

69. Qiu, X.; Guan, X.; Liu, W.; Zhang, Y. DAL-1 attenuates epithelial to mesenchymal transition and metastasis by suppressing HSPA5 expression in non-small cell lung cancer. Oncol Rep 2017, 38, 3103-3113, doi:10.3892/or.2017.6000.

70. Shu, C.W.; Sun, F.C.; Cho, J.H.; Lin, C.C.; Liu, P.F.; Chen, P.Y.; Chang, M.D.; Fu, H.W.; Lai, Y.K. GRP78 and Raf-1 cooperatively confer resistance to endoplasmic reticulum stress-induced apoptosis. J Cell Physiol 2008, 215, 627-635, doi:10.1002/jcp.21340.

71. Reddy, R.K.; Mao, C.; Baumeister, P.; Austin, R.C.; Kaufman, R.J.; Lee, A.S. Endoplasmic reticulum chaperone protein GRP78 protects cells from apoptosis induced by topoisomerase inhibitors: role of ATP binding site in suppression of caspase-7 activation. J Biol Chem 2003, 278, 20915-20924, doi:10.1074/jbc.M212328200.

72. Zhu, H.; Cao, X.; Cai, X.; Tian, Y.; Wang, D.; Qi, J.; Teng, Z.; Lu, G.; Ni, Q.; Wang, S.; et al. Pifithrin-μ incorporated in gold nanoparticle amplifies pro-apoptotic unfolded protein response cascades to potentiate synergistic glioblastoma therapy. Biomaterials 2020, 232, 119677, doi:10.1016/j.biomaterials.2019.119677.

73. Verras, M.; Papandreou, I.; Lim, A.L.; Denko, N.C. Tumor hypoxia blocks Wnt processing and secretion through the induction of endoplasmic reticulum stress. Mol Cell Biol 2008, 28, 7212-7224, doi:10.1128/MCB.00947-08.

74. Aran, G.; Sanjurjo, L.; Bárcena, C.; Simon-Coma, M.; Téllez, É.; Vázquez-Vitali, M.; Garrido, M.; Guerra, L.; Díaz, E.; Ojanguren, I.; et al. CD5L is upregulated in hepatocellular carcinoma and promotes liver cancer cell proliferation and antiapoptotic responses by binding to HSPA5 (GRP78). FASEB J 2018, 32, 3878-3891, doi:10.1096/fj.201700941RR.

75. Pi, L.; Li, X.; Song, Q.; Shen, Y.; Lu, X.; DI, B. Knockdown of glucose-regulated protein 78 abrogates chemoresistance of hypopharyngeal carcinoma cells to cisplatin induced by unfolded protein in response to severe hypoxia. Oncol Lett 2014, 7, 685-692, doi:10.3892/ol.2013.1753.

76. Chen, Y.; Mi, Y.; Zhang, X.; Ma, Q.; Song, Y.; Zhang, L.; Wang, D.; Xing, J.; Hou, B.; Li, H.; et al. Dihydroartemisinin-induced unfolded protein response feedback attenuates ferroptosis via PERK/ATF4/HSPA5 pathway in glioma cells. J Exp Clin Cancer Res 2019, 38, 402, doi:10.1186/s13046-019-1413-7.

77. Kern, J.; Untergasser, G.; Zenzmaier, C.; Sarg, B.; Gastl, G.; Gunsilius, E.; Steurer, M. GRP-78 secreted by tumor cells blocks the antiangiogenic activity of bortezomib. Blood 2009, 114, 3960-3967, doi:10.1182/blood-2009-03-209668.

78. Li, Z. Glucose regulated protein 78: a critical link between tumor microenvironment and cancer hallmarks. Biochim Biophys Acta 2012, 1826, 13-22, doi:10.1016/j.bbcan.2012.02.001.

79. Chou, C.W.; Yang, R.Y.; Chan, L.C.; Li, C.F.; Sun, L.; Lee, H.H.; Lee, P.C.; Sher, Y.P.; Ying, H.; Hung, M.C. Erratum: The stabilization of PD-L1 by the endoplasmic reticulum stress protein GRP78 in triple-negative breast cancer. Am J Cancer Res 2020, 10, 3507.

80. Oida, T.; Weiner, H.L. Overexpression of TGF-ß 1 gene induces cell surface localized glucose-regulated protein 78-associated latency-associated peptide/TGF-ß. J Immunol 2010, 185, 3529-3535, doi:10.4049/jimmunol.0904121.

81. Meng, J.; Chen, S.; Lei, Y.Y.; Han, J.X.; Zhong, W.L.; Wang, X.R.; Liu, Y.R.; Gao, W.F.; Zhang, Q.; Tan, Q.; et al. Hsp90β promotes aggressive vasculogenic mimicry via epithelial-mesenchymal transition in hepatocellular carcinoma. Oncogene 2019, 38, 228-243, doi:10.1038/s41388-018-0428-4.

82. Wei, P.L.; Huang, C.Y.; Tai, C.J.; Batzorig, U.; Cheng, W.L.; Hunag, M.T.; Chang, Y.J. Glucose-regulated protein 94 mediates metastasis by CCT8 and the JNK pathway in hepatocellular carcinoma. Tumour Biol 2016, 37, 8219-8227, doi:10.1007/s13277-015-4669-3.

83. Huang, C.Y.; Batzorig, U.; Cheng, W.L.; Huang, M.T.; Chen, W.; Wei, P.L.; Chang, Y.J. Glucose-regulated protein 94 mediates cancer progression via AKT and eNOS in hepatocellular carcinoma. Tumour Biol 2016, 37, 4295-4304, doi:10.1007/s13277-015-4254-9.

84. Suzuki, S.; Kulkarni, A.B. Extracellular heat shock protein HSP90beta secreted by MG63 osteosarcoma cells inhibits activation of latent TGF-beta1. Biochem Biophys Res Commun 2010, 398, 525-531, doi:10.1016/j.bbrc.2010.06.112.

85. Mahajan, N.P.; Whang, Y.E.; Mohler, J.L.; Earp, H.S. Activated tyrosine kinase Ack1 promotes prostate tumorigenesis: role of Ack1 in polyubiquitination of tumor suppressor Wwox. Cancer Res 2005, 65, 10514-10523, doi:10.1158/0008-5472.CAN-05-1127.

86. Ghosh, S.; Shinogle, H.E.; Galeva, N.A.; Dobrowsky, R.T.; Blagg, B.S. Endoplasmic Reticulum-resident Heat Shock Protein 90 (HSP90) Isoform Glucose-regulated Protein 94 (GRP94) Regulates Cell Polarity and Cancer Cell Migration by Affecting Intracellular Transport. J Biol Chem 2016, 291, 8309-8323, doi:10.1074/jbc.M115.688374.

87. Hu, T.; Xie, N.; Qin, C.; Wang, J.; You, Y. Glucose-regulated protein 94 is a novel glioma biomarker and promotes the aggressiveness of glioma via Wnt/β-catenin signaling pathway. Tumour Biol 2015, 36, 9357-9364, doi:10.1007/s13277-015-3635-4.

88. Wu, B.; Chu, X.; Feng, C.; Hou, J.; Fan, H.; Liu, N.; Li, C.; Kong, X.; Ye, X.; Meng, S. Heat shock protein gp96 decreases p53 stability by regulating Mdm2 E3 ligase activity in liver cancer. Cancer Lett 2015, 359, 325-334, doi:10.1016/j.canlet.2015.01.034.

89. Graner, M.W. HSP90 and Immune Modulation in Cancer. Adv Cancer Res 2016, 129, 191-224, doi:10.1016/bs.acr.2015.10.001.

90. Duan, X.F.; Xin, Y.W. Overexpression of molecule GRP94 favors tumor progression in lung adenocarcinoma by interaction with regulatory T cells. Thorac Cancer 2020, 11, 704-712, doi:10.1111/1759-7714.13321.

91. Lu, W.W.; Zhang, H.; Li, Y.M.; Ji, F. Gastric cancer-derived heat shock protein-gp96 peptide complex enhances dendritic cell activation. World J Gastroenterol 2017, 23, 4390-4398, doi:10.3748/wjg.v23.i24.4390.

92. Meng, J.; Liu, Y.; Han, J.; Tan, Q.; Chen, S.; Qiao, K.; Zhou, H.; Sun, T.; Yang, C. Hsp90β promoted endothelial cell-dependent tumor angiogenesis in hepatocellular carcinoma. Mol Cancer 2017, 16, 72, doi:10.1186/s12943-017-0640-9.

93. Li, S.; Li, J.; Hu, T.; Zhang, C.; Lv, X.; He, S.; Yan, H.; Tan, Y.; Wen, M.; Lei, M.; et al. Bcl-2 overexpression contributes to laryngeal carcinoma cell survival by forming a complex with Hsp90β. Oncol Rep 2017, 37, 849-856, doi:10.3892/or.2016.5295.

94. Hunter, M.C.; O'Hagan, K.L.; Kenyon, A.; Dhanani, K.C.; Prinsloo, E.; Edkins, A.L. Hsp90 binds directly to fibronectin (FN) and inhibition reduces the extracellular fibronectin matrix in breast cancer cells. PLoS One 2014, 9, e86842, doi:10.1371/journal.pone.0086842.

95. Hamamoto, R.; Toyokawa, G.; Nakakido, M.; Ueda, K.; Nakamura, Y. SMYD2-dependent HSP90 methylation promotes cancer cell proliferation by regulating the chaperone complex formation. Cancer Lett 2014, 351, 126-133, doi:10.1016/j.canlet.2014.05.014.

96. Wang, H.; Deng, G.; Ai, M.; Xu, Z.; Mou, T.; Yu, J.; Liu, H.; Wang, S.; Li, G. Hsp90ab1 stabilizes LRP5 to promote epithelial-mesenchymal transition via activating of AKT and Wnt/β-catenin signaling pathways in gastric cancer progression. Oncogene 2019, 38, 1489-1507, doi:10.1038/s41388-018-0532-5.

97. Bian, Y.; Guo, J.; Qiao, L.; Sun, X. miR-3189-3p Mimics Enhance the Effects of S100A4 siRNA on the Inhibition of Proliferation and Migration of Gastric Cancer Cells by Targeting CFL2. Int J Mol Sci 2018, 19, doi:10.3390/ijms19010236.

98. Wo, Q.; Zhang, D.; Hu, L.; Lyu, J.; Xiang, F.; Zheng, W.; Shou, J.; Qi, X. Long noncoding RNA SOX2-OT facilitates prostate cancer cell proliferation and migration via miR-369-3p/CFL2 axis. Biochem Biophys Res Commun 2019, 520, 586-593, doi:10.1016/j.bbrc.2019.09.108.

99. Li, P.; Wang, Z.; Li, S.; Wang, L. Circ_0006404 Accelerates Prostate Cancer Progression Through Regulating miR-1299/CFL2 Signaling. Onco Targets Ther 2021, 14, 83-95, doi:10.2147/OTT.S277831.

100. Deng, B.; Fang, J.; Zhang, X.; Qu, L.; Cao, Z.; Wang, B. Role of gelsolin in cell proliferation and invasion of human hepatocellular carcinoma cells. Gene 2015, 571, 292-297, doi:10.1016/j.gene.2015.06.083.

101. Zhang, Y.; Luo, X.; Lin, J.; Fu, S.; Feng, P.; Su, H.; He, X.; Liang, X.; Liu, K.; Deng, W. Gelsolin Promotes Cancer Progression by Regulating Epithelial-Mesenchymal Transition in Hepatocellular Carcinoma and Correlates with a Poor Prognosis. J Oncol 2020, 2020, 1980368, doi:10.1155/2020/1980368.

102. Ma, X.; Sun, W.; Shen, J.; Hua, Y.; Yin, F.; Sun, M.; Cai, Z. Gelsolin promotes cell growth and invasion through the upregulation of p-AKT and p-P38 pathway in osteosarcoma. Tumour Biol 2016, 37, 7165-7174, doi:10.1007/s13277-015-4565-x.

103. Wang, P.W.; Abedini, M.R.; Yang, L.X.; Ding, A.A.; Figeys, D.; Chang, J.Y.; Tsang, B.K.; Shieh, D.B. Gelsolin regulates cisplatin sensitivity in human head-and-neck cancer. Int J Cancer 2014, 135, 2760-2769, doi:10.1002/ijc.28928.

104. Zhou, Y.; Deng, X.; Ma, X.; Zang, N.; Li, H.; Li, G.; Li, D.; Li, C.; Huang, W.; He, M. Cellular transcriptomics: gelsolin negatively regulates the expression of apoptosis-associated genes and inhibits apoptosis in hepatocarcinoma cells. Int J Clin Exp Pathol 2015, 8, 13871-13885.

105. Jackson, H.W.; Hojilla, C.V.; Weiss, A.; Sanchez, O.H.; Wood, G.A.; Khokha, R. Timp3 deficient mice show resistance to developing breast cancer. PLoS One 2015, 10, e0120107, doi:10.1371/journal.pone.0120107.

106. Rauscher, B.; Heigwer, F.; Henkel, L.; Hielscher, T.; Voloshanenko, O.; Boutros, M. Toward an integrated map of genetic interactions in cancer cells. Mol Syst Biol 2018, 14, e7656, doi:10.15252/msb.20177656.

107. Qin, Y.; Zhao, L.; Wang, X.; Tong, D.; Hoover, C.; Wu, F.; Liu, Y.; Wang, L.; Liu, L.; Ni, L.; et al. MeCP2 regulated glycogenes contribute to proliferation and apoptosis of gastric cancer cells. Glycobiology 2017, 27, 306-317, doi:10.1093/glycob/cwx006.

108. Wang, J.M.; Jiang, J.Y.; Zhang, D.L.; Du, X.; Wu, T.; Du, Z.X. HYOU1 facilitates proliferation, invasion and glycolysis of papillary thyroid cancer via stabilizing LDHB mRNA. J Cell Mol Med 2021, 25, 4814-4825, doi:10.1111/jcmm.16453.

109. Lee, M.; Song, Y.; Choi, I.; Lee, S.Y.; Kim, S.; Kim, S.H.; Kim, J.; Seo, H.R. Expression of HYOU1 via Reciprocal Crosstalk between NSCLC Cells and HUVECs Control Cancer Progression and Chemoresistance in Tumor Spheroids. Mol Cells 2021, 44, 50-62, doi:10.14348/molcells.2020.0212.

110. Namba, T.; Hoshino, T.; Tanaka, K.; Tsutsumi, S.; Ishihara, T.; Mima, S.; Suzuki, K.; Ogawa, S.; Mizushima, T. Up-regulation of 150-kDa oxygen-regulated protein by celecoxib in human gastric carcinoma cells. Mol Pharmacol 2007, 71, 860-870, doi:10.1124/mol.106.027698.

111. Miyagi, T.; Hori, O.; Koshida, K.; Egawa, M.; Kato, H.; Kitagawa, Y.; Ozawa, K.; Ogawa, S.; Namiki, M. Antitumor effect of reduction of 150-kDa oxygen-regulated protein expression on human prostate cancer cells. Int J Urol 2002, 9, 577-585, doi:10.1046/j.1442-2042.2002.00519.x.

112. Li, X.; Zhang, N.X.; Ye, H.Y.; Song, P.P.; Chang, W.; Chen, L.; Wang, Z.; Zhang, L.; Wang, N.N. HYOU1 promotes cell growth and metastasis via activating PI3K/AKT signaling in epithelial ovarian cancer and predicts poor prognosis. Eur Rev Med Pharmacol Sci 2019, 23, 4126-4135, doi:10.26355/eurrev_201901_17914.

113. Asahi, H.; Koshida, K.; Hori, O.; Ogawa, S.; Namiki, M. Immunohistochemical detection of the 150-kDa oxygen-regulated protein in bladder cancer. BJU Int 2002, 90, 462-466, doi:10.1046/j.1464-410x.2002.02915.x.

114. van der Hoeven, D.; Cho, K.J.; Zhou, Y.; Ma, X.; Chen, W.; Naji, A.; Montufar-Solis, D.; Zuo, Y.; Kovar, S.E.; Levental, K.R.; et al. Sphingomyelin Metabolism Is a Regulator of K-Ras Function. Mol Cell Biol 2018, 38, doi:10.1128/MCB.00373-17.

115. Heering, J.; Weis, N.; Holeiter, M.; Neugart, F.; Staebler, A.; Fehm, T.N.; Bischoff, A.; Schiller, J.; Duss, S.; Schmid, S.; et al. Loss of the ceramide transfer protein augments EGF receptor signaling in breast cancer. Cancer Res 2012, 72, 2855-2866, doi:10.1158/0008-5472.CAN-11-3069.

116. Schultz, J.; Lorenz, P.; Gross, G.; Ibrahim, S.; Kunz, M. MicroRNA let-7b targets important cell cycle molecules in malignant melanoma cells and interferes with anchorage-independent growth. Cell Res 2008, 18, 549-557, doi:10.1038/cr.2008.45.

117. Wang, S.; Wu, Y.; Xu, Y.; Tang, X. miR-10b promoted melanoma progression through Wnt/β-catenin pathway by repressing ITCH expression. Gene 2019, 710, 39-47, doi:10.1016/j.gene.2019.05.043.

118. Zhang, Z.; Zhang, S.; Ma, P.; Jing, Y.; Peng, H.; Gao, W.Q.; Zhuang, G. Lin28B promotes melanoma growth by mediating a microRNA regulatory circuit. Carcinogenesis 2015, 36, 937-945, doi:10.1093/carcin/bgv085.

119. Koetz-Ploch, L.; Hanniford, D.; Dolgalev, I.; Sokolova, E.; Zhong, J.; Díaz-Martínez, M.; Bernstein, E.; Darvishian, F.; Flaherty, K.T.; Chapman, P.B.; et al. MicroRNA-125a promotes resistance to BRAF inhibitors through suppression of the intrinsic apoptotic pathway. Pigment Cell Melanoma Res 2017, 30, 328-338, doi:10.1111/pcmr.12578.

120. Yang, X.; Zhao, H.; Yang, J.; Ma, Y.; Liu, Z.; Li, C.; Wang, T.; Yan, Z.; Du, N. MiR-150-5p regulates melanoma proliferation, invasion and metastasis via SIX1-mediated Warburg Effect. Biochem Biophys Res Commun 2019, 515, 85-91, doi:10.1016/j.bbrc.2019.05.111.

121. Luan, W.; Li, R.; Liu, L.; Ni, X.; Shi, Y.; Xia, Y.; Wang, J.; Lu, F.; Xu, B. Long non-coding RNA HOTAIR acts as a competing endogenous RNA to promote malignant melanoma progression by sponging miR-152-3p. Oncotarget 2017, 8, 85401-85414, doi:10.18632/oncotarget.19910.

122. Latchana, N.; Ganju, A.; Howard, J.H.; Carson, W.E. MicroRNA dysregulation in melanoma. Surg Oncol 2016, 25, 184-189, doi:10.1016/j.suronc.2016.05.017.

123. Zhang, L.; He, X.; Li, F.; Pan, H.; Huang, X.; Wen, X.; Zhang, H.; Li, B.; Ge, S.; Xu, X.; et al. The miR-181 family promotes cell cycle by targeting CTDSPL, a phosphatase-like tumor suppressor in uveal melanoma. J Exp Clin Cancer Res 2018, 37, 15, doi:10.1186/s13046-018-0679-5.

124. Gyukity-Sebestyén, E.; Harmati, M.; Dobra, G.; Németh, I.B.; Mihály, J.; Zvara, Á.; Hunyadi-Gulyás, É.; Katona, R.; Nagy, I.; Horváth, P.; et al. Melanoma-Derived Exosomes Induce PD-1 Overexpression and Tumor Progression via Mesenchymal Stem Cell Oncogenic Reprogramming. Front Immunol 2019, 10, 2459, doi:10.3389/fimmu.2019.02459.

125. Nagpal, N.; Kulshreshtha, R. miR-191: an emerging player in disease biology. Front Genet 2014, 5, 99, doi:10.3389/fgene.2014.00099.

126. Mueller, D.W.; Bosserhoff, A.K. MicroRNA miR-196a controls melanoma-associated genes by regulating HOX-C8 expression. Int J Cancer 2011, 129, 1064-1074, doi:10.1002/ijc.25768.

127. Pencheva, N.; Tran, H.; Buss, C.; Huh, D.; Drobnjak, M.; Busam, K.; Tavazoie, S.F. Convergent multi-miRNA targeting of ApoE drives LRP1/LRP8-dependent melanoma metastasis and angiogenesis. Cell 2012, 151, 1068-1082, doi:10.1016/j.cell.2012.10.028.

128. Bustos, M.A.; Ono, S.; Marzese, D.M.; Oyama, T.; Iida, Y.; Cheung, G.; Nelson, N.; Hsu, S.C.; Yu, Q.; Hoon, D.S.B. MiR-200a Regulates CDK4/6 Inhibitor Effect by Targeting CDK6 in Metastatic Melanoma. J Invest Dermatol 2017, 137, 1955-1964, doi:10.1016/j.jid.2017.03.039.

129. Zhou, W.J.; Wang, H.Y.; Zhang, J.; Dai, H.Y.; Yao, Z.X.; Zheng, Z.; Meng-Yan, S.; Wu, K. NEAT1/miR-200b-3p/SMAD2 axis promotes progression of melanoma. Aging (Albany NY) 2020, 12, 22759-22775, doi:10.18632/aging.103909.

130. Sahranavardfard, P.; Firouzi, J.; Azimi, M.; Khosravani, P.; Heydari, R.; Emami Razavi, A.; Dorraj, M.; Keighobadi, F.; Ebrahimi, M. MicroRNA-203 reinforces stemness properties in melanoma and augments tumorigenesis in vivo. J Cell Physiol 2019, 234, 20193-20205, doi:10.1002/jcp.28619.

131. Noman, M.Z.; Buart, S.; Romero, P.; Ketari, S.; Janji, B.; Mari, B.; Mami-Chouaib, F.; Chouaib, S. Hypoxia-inducible miR-210 regulates the susceptibility of tumor cells to lysis by cytotoxic T cells. Cancer Res 2012, 72, 4629-4641, doi:10.1158/0008-5472.CAN-12-1383.

132. Martin del Campo, S.E.; Latchana, N.; Levine, K.M.; Grignol, V.P.; Fairchild, E.T.; Jaime-Ramirez, A.C.; Dao, T.V.; Karpa, V.I.; Carson, M.; Ganju, A.; et al. MiR-21 enhances melanoma invasiveness via inhibition of tissue inhibitor of metalloproteinases 3 expression: in vivo effects of MiR-21 inhibitor. PLoS One 2015, 10, e0115919, doi:10.1371/journal.pone.0115919.

133. Melnik, B.C. MiR-21: an environmental driver of malignant melanoma? J Transl Med 2015, 13, 202, doi:10.1186/s12967-015-0570-5.

134. Yang, Z.; Liao, B.; Xiang, X.; Ke, S. miR-21-5p promotes cell proliferation and G1/S transition in melanoma by targeting CDKN2C. FEBS Open Bio 2020, 10, 752-760, doi:10.1002/2211-5463.12819.

135. Knoll, S.; Fürst, K.; Kowtharapu, B.; Schmitz, U.; Marquardt, S.; Wolkenhauer, O.; Martin, H.; Pützer, B.M. E2F1 induces miR-224/452 expression to drive EMT through TXNIP downregulation. EMBO Rep 2014, 15, 1315-1329, doi:10.15252/embr.201439392.

136. Lunavat, T.R.; Cheng, L.; Einarsdottir, B.O.; Olofsson Bagge, R.; Veppil Muralidharan, S.; Sharples, R.A.; Lässer, C.; Gho, Y.S.; Hill, A.F.; Nilsson, J.A.; et al. BRAF. Proc Natl Acad Sci U S A 2017, 114, E5930-E5939, doi:10.1073/pnas.1705206114.

137. Lazaridou, M.F.; Massa, C.; Handke, D.; Mueller, A.; Friedrich, M.; Subbarayan, K.; Tretbar, S.; Dummer, R.; Koelblinger, P.; Seliger, B. Identification of microRNAs Targeting the Transporter Associated with Antigen Processing TAP1 in Melanoma. J Clin Med 2020, 9, doi:10.3390/jcm9092690.

138. Li, Y.; Zhang, J.; Liu, Y.; Zhang, B.; Zhong, F.; Wang, S.; Fang, Z. MiR-30a-5p confers cisplatin resistance by regulating IGF1R expression in melanoma cells. BMC Cancer 2018, 18, 404, doi:10.1186/s12885-018-4233-9.

139. Gaziel-Sovran, A.; Segura, M.F.; Di Micco, R.; Collins, M.K.; Hanniford, D.; Vega-Saenz de Miera, E.; Rakus, J.F.; Dankert, J.F.; Shang, S.; Kerbel, R.S.; et al. miR-30b/30d regulation of GalNAc transferases enhances invasion and immunosuppression during metastasis. Cancer Cell 2011, 20, 104-118, doi:10.1016/j.ccr.2011.05.027.

140. Paschen, A.; Baingo, J.; Schadendorf, D. Expression of stress ligands of the immunoreceptor NKG2D in melanoma: regulation and clinical significance. Eur J Cell Biol 2014, 93, 49-54, doi:10.1016/j.ejcb.2014.01.009.

141. Bai, X.; Yang, M.; Xu, Y. MicroRNA-373 promotes cell migration via targeting salt-inducible kinase 1 expression in melanoma. Exp Ther Med 2018, 16, 4759-4764, doi:10.3892/etm.2018.6784.

142. Sun, Y.; Li, C.; Lu, Q.; Jiang, H.; Zhu, M.; Huang, G.; Wang, T. Integrative Analysis of lncRNA-mRNA Profile Reveals Potential Predictors for SAPHO Syndrome. Front Genet 2021, 12, 684520, doi:10.3389/fgene.2021.684520.
